# Genome Functional Analysis of the Psychrotrophic Lignin-Degrading Bacterium *Arthrobacter* sp. C2 and the Role of DyP in Catalyzing Lignin Degradation

**DOI:** 10.3389/fmicb.2022.921549

**Published:** 2022-07-13

**Authors:** Cheng Jiang, Haohao Yan, Xiaohui Shen, Yuting Zhang, Yue Wang, Shanshan Sun, Hanyi Jiang, Hailian Zang, Xinyue Zhao, Ning Hou, Ziwei Li, Liwen Wang, Hanjun Wang, Chunyan Li

**Affiliations:** ^1^College of Resources and Environment, Northeast Agricultural University, Harbin, China; ^2^College of Life Science and Resources and Environment, Yichun University, Yichun, China

**Keywords:** psychrotrophic lignin-degrading bacterium, *Arthrobacter*, metabolic mechanism, whole genome, *DyP*

## Abstract

In the cold regions of China, lignin-rich corn straw accumulates at high levels due to low temperatures. The application of psychrotrophic lignin-degrading bacteria should be an effective means of overcoming the low-temperature limit for lignin degradation and promoting the utilization of corn straw. However, this application is limited by the lack of suitable strains for decomposition of lignin; furthermore, the metabolic mechanism of psychrotrophic lignin-degrading bacteria is unclear. Here, the whole genome of the psychrotrophic lignin-degrading bacterium *Arthrobacter* sp. C2, isolated in our previous work, was sequenced. Comparative genomics revealed that C2 contained unique genes related to lignin degradation and low-temperature adaptability. DyP may participate in lignin degradation and may be a cold-adapted enzyme. Moreover, DyP was proven to catalyze lignin Cα-Cβ bond cleavage. Deletion and complementation of the *DyP* gene verified its ability to catalyze the first-step reaction of lignin degradation. Comparative transcriptomic analysis revealed that the transcriptional expression of the *DyP* gene was upregulated, and the genetic compensation mechanism allowed C2Δ*DyP* to degrade lignin, which provided novel insights into the survival strategy of the psychrotrophic mutant strain C2Δ*dyP*. This study improved our understanding of the metabolic mechanism of psychrotrophic lignin-degrading bacteria and provided potential application options for energy-saving production using cold-adapted lignin-degrading enzymes.

## Graphical Abstract

The psychrotrophic lignin-degrading *Arthrobacter* sp. C2 strain exhibited lignin degradation ability and low temperature adaptability. The cold-adapted DyP enzyme of C2 was involved in the catalytic cleavage of lignin Cα-Cβ bonds as a key lignin-degrading enzyme. Comparative transcriptomic analysis showed that the induced transcriptional expression of DyP genes increased on lignin medium, and the mechanism of genetic compensation explained the survival strategy of the mutant strain C2ΔDyP. 
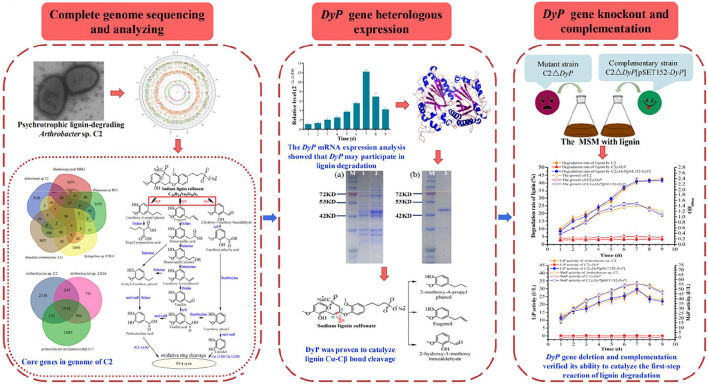

## Introduction

Lignin is one of the most abundant natural polymers and is an important renewable source of valuable products, such as fuels, chemicals and biobased materials. The total amount of available lignin in the biosphere has reached 300 billion tons, increasing by approximately 20 billion tons annually (Becker and Wittmann, 2019). Over 80% of the biosphere is cold, and a large proportion of lignin comes from low-temperature areas, including seasonally cold environments, for example, 55% of Russia and Canada, 85% of Alaska, and 20% of China (Zhang et al., 2015). In China, as an important source of lignin, crop straw is abundant, with a yield of 800 million tons annually, including approximately 222 million tons of corn straw output (Yang et al., 2016). The yield of corn straw in northeast China accounts for 31% that in the whole country, reaching 68.5 million tons (Qinggeer et al., 2016). However, due to the high latitude, long winter, 5–6 months of freezing conditions and low temperatures (He et al., 2009), most of the corn straw is burned, limiting the implementation of better treatment strategies such as straw return to the field, resulting in not only severe air pollution but also the waste of lignin resources (Yang et al., 2016). Finding effective ways to tackle lignin degradation is the key to utilizing lignin resources.

Several strategies for lignin treatment have emerged, including physical, chemical and microbial methods (Ponnusamy et al., 2019). Microbial methods present the advantages of low cost and environmental friendliness (Abdellah et al., 2021). Recently, a study on the contribution of bacteria to delignification and lignocellulose degradation in forest soils revealed that the main microbial group that decomposes lignin is bacteria (Wilhelm et al., 2018). Bacteria can serve as useful tools for lignin degradation due to their extremely high environmental adaptability, biochemical versatility and enzyme production ability (Zheng et al., 2013). Lignin-degrading bacteria such as *Pseudomonas*, *Bacillus*, *Streptomyces*, *Rhodococcus*, *Comamonas*, *Enterobacter*, *Klebsiella*, *Oceanimonas*, and *Pandoraea* species have been discovered in different environments (Zheng et al., 2013; Palazzolo and Kurina-Sanz, 2016). Among these species, *Pandoraea* sp. strain B-6 had the ability to degrade lignin at 30°C, with a maximum degradation rate of 39.9% (Zheng et al., 2013). *Streptomyces* spp. strains F-6 and F-7 isolated from forest soil showed lignin degradation rates of 37.6 and 38.1% at 30°C, respectively (Yang et al., 2012). When *Aneurinibacillus aneurinilyticus* ITRC S7 was cultured for 6 days at 30°C and pH 7.6, it exhibited a lignin degradation rate of 43% (Raj et al., 2007). However, the abovementioned lignin-degrading bacteria include few psychrotrophic microorganisms, which may be candidates for the degradation of lignin at low temperatures. The metabolic mechanisms of psychrotrophic strains need to be further explored.

Complete genome analysis is considered to be an effective means of exploring the metabolic mechanisms of psychrotrophic microorganisms, facilitating improved utilization of microbial resources and the exploitation of cold-adapted enzymes (An et al., 2020). To date, only four strains of lignin-degrading bacteria have been subjected to whole-genome sequencing: *Rhodococcus jostii* RHA1, *Raoultella ornithinolytica* S12, *Sphingobium* sp. SYK-6, and *Halomonas* sp. KO116. However, these bacteria are mesophilic strains.

The degradation of lignin by microorganisms depends on the activity of ligninolytic enzymes, including manganese peroxidases (MnPs), lignin peroxidases (LiPs), versatile peroxidases (VPs), laccases (Lacs) and dye-decolorizing peroxidases (DyPs) (Becker and Wittmann, 2019). Among them, DyPs represent a relatively recently discovered peroxidase family that was first identified in *Bjerkandera adusta* in 1999 (Kim and Shoda, 1999) and was then found in other fungi and some bacteria (Sugano, 2009). DyP has a wide range of substrates (Lončar et al., 2016), and its catalytic mechanism is similar to that of lignin peroxidase and manganese peroxidase (Sugano, 2009). DyP has been found mainly in bacteria and may play an important role in lignin degradation (Colpa et al., 2014). Bacterial DyP could represent a bacterial counterpart of fungal lignin peroxidase (Lončar et al., 2016). However, research on DyP has focused mainly on its enzymatic characteristics, and there is a lack of information about the catalytic mechanism of DyP toward lignin. TcDyP from *Thermomonospora curvata* showed catalytic activity for phenolic lignin at the optimal temperature of 30°C (Chen et al., 2015). The enzymatic properties of the DyP peroxidase RaoPrx in the *R. ornithinolytica* OKOH-1 strain indicated that this enzyme exhibited optimum activity at 50°C (Falade et al., 2019). The DyPB of *R. jostii* RHA1 could cleave the Cα-Cβ bond of lignin at 30°C (Ahmad et al., 2011). However, these DyPs showed optimal enzyme activity at mesophilic temperature, and there is a lack of research on cold-adapted DyPs. Cold-adapted enzymes are especially useful for the development of low-temperature detergents, food and industrial biocatalytic enzymes, and bioremediation agents that are suitable for cold regions. However, little is known about cold-adapted lignin-degrading enzymes, especially regarding their catalytic mechanisms under low temperature.

In the author’s previous research, a psychrotrophic *Arthrobacter* strain (*Arthrobacter* sp. C2) with the ability to degrade lignin at 15°C was isolated. In the present study, the complete genome sequence, genomic characteristics, genetic basis of lignin degradation, and environmental adaptation were analyzed for this bacterium. The role of the *DyP* gene in lignin degradation in *Arthrobacter* sp. C2 was confirmed by analysis of *DyP* mRNA expression and enzymatic properties and by generating *DyP* gene knockout and complementation strains. The results of this study will facilitate the application of psychrotrophic bacterial strains and cold-adapted enzyme resources and provide new insight to promote the utilization of lignin resources in cold regions of the biosphere.

## Materials and Methods

### Bacterial Strain, Plasmids, and Medium

The psychrotrophic lignin-degrading *Arthrobacter* sp. C2 strain was isolated in the author’s previous study. The growth medium for C2 was lignin mineral salt medium (L-MSM), which consisted of 3.0 g of sodium lignin sulfonate, 1.4 g of (NH_4_)_2_SO_4_, 0.5 g of MgSO_4_⋅7H_2_O, 2.0 g of K_2_HPO_4_, 0.3 g of CaCl_2_, 0.005 g of FeSO_4_⋅7H_2_O, 0.0016 g of MnSO_4_, 0.0017 g of ZnCl_2_, and 0.0017 g of CoCl_2_ (pH 7.0). The pET-28a(+) plasmid was used as the expression vector. *Escherichia coli* DH5α and BL21(DE3) were used for gene cloning and expression. The plasmids pUCm-T and pUC57 were used as the cloning vectors. The above plasmids and strains were purchased from Sangon Biotech Co., Ltd., China. The pSET152 plasmid (Ke Lei Biological Technology Co., Ltd., China) was used as the complementation vector. All chemicals and biochemical reagents were purchased from Tianjin Kemiou Chemical Reagent Co., Ltd., China. All enzymes used for DNA manipulation in this work were provided by TaKaRa Biotechnology (Dalian, China).

### Whole-Genome Sequencing of Psychrotrophic *Arthrobacter* sp. C2

The total genomic DNA of C2 was extracted by using a Qiagen DNA extraction kit (Hilden, Germany). The whole genome of C2 was sequenced and assembled by Personal Biotechnology Co. Genome sequencing of C2 was performed using both Illumina MiSeq II and PacBio RS Ø sequencing technologies and Pacific Biosciences platform technology, and Glimmer 3.0 software was used for gene prediction and annotation (Zang et al., 2020; Hou et al., 2021). Genes were predicted in the C2 genome, and annotation was performed in the GO, COG, Nr, KEGG, and SwissProt databases. A circular genome map was generated with CGView software (Zhao Q. et al., 2017). Prediction of RNA and genomic islands (GIs) was carried out as described by Liu et al. (2017). Genes related to basic metabolism, lignin degradation, and environmental adaptation in the C2 genome were identified via a search.

### Genomic Comparisons With Other Lignin-Degrading Bacteria and Other *Arthrobacter* Strains

Genomic features were compared among strain C2 and other lignin-degrading and *Arthrobacter* sp. bacteria for which whole-genome sequencing has been completed. Then, the BLAST tool was used to compare the similarity of the amino acid sequences of lignin-degrading enzymes. The evolutionary relationships of *Arthrobacter* sp. C2 were assessed by phylogenetic analysis. The lignin-degrading genes and low-temperature adaptation genes in the genomes of the above lignin-degrading bacteria and *Arthrobacter* sp. bacteria were compared with those of strain C2.

### Detection of mRNA Expression of the Key Lignin Degradation Gene *DyP* by RT–qPCR

To measure the expression of the *DyP* gene during lignin degradation, the dynamic changes in the *DyP* gene at the transcriptional level were detected by RT–qPCR with 16S rDNA as an internal reference. The transcription level of the *DyP* gene was evaluated by the 2 ^–^^ΔΔ*CT*^ method (Li et al., 2020). Reactions were carried out in a 20 μL reaction volume. The reaction conditions were as follows: 95°C for 3 min, followed by 40 cycles of 95°C for 5 s, 60°C for 30 s, and 72°C for 30 s. Each sample was replicated three times. In addition, the primer sequences and the amplification reaction system are shown in Supplementary Tables 1, 2, respectively.

### Gene Expression and Purification of Recombinant DyP

The genomic DNA of C2 was used as a template for gene-specific primed PCR amplification of the *DyP* gene. The gene encoding *DyP* was cloned into pET-28a(+), and the recombinant plasmid pET28(+)-*DyP* was transformed into *E. coli* BL21(DE3). The expression of DyP was induced by the addition of 0.2 mM IPTG when the optical density reached approximately 0.6 at 600 nm. The cells were collected by centrifugation at 6000 rpm and 4°C for 30 min and resuspended in 5 mL of phosphate-buffered saline (PBS) at pH 7.2. The suspension was subjected to ultrasonication with a SCIENTZ-IID ultrasonic homogenizer (Ningbo Scientz Biotechnology Co., Ltd. Zhejiang Province, China). The resulting cell lysate was centrifuged at 4°C for 30 min at 12,000 rpm. Then, SDS–PAGE was performed to analyze the supernatant. The protein was purified with Ni-NTA agarose (GE Healthcare, United States) according to the methods described by Zang et al. (2020). The molecular mass of the DyP protein was determined by SDS–PAGE. The purified DyP protein concentration was determined by the Bradford method (Zang et al., 2020).

### Lignin Degradation Properties of the Recombinant DyP Protein

The reaction was performed at 15°C for 24 h by adding 1 mL of purified DyP protein to 15 mL of PBS containing 3 g/L sodium lignin sulfonate. After the reaction was performed, an equal volume of ethyl acetate was added for extraction three times, and the obtained organic phase was evaporated and concentrated by vacuum rotation. Finally, the sample was filtered through a 0.22-μm filter membrane for GC–MS analysis. The details of the assay were described by Jiang et al. (2019).

### Biochemical Characterization of Recombinant DyP

The optimal temperature for the DyP catalytic reaction was determined by performing the reaction in a water bath at 5–60°C. To determine the effect of temperature on the stability of DyP, the enzyme was incubated in a water bath for 2 h at different temperatures within the range of 5–60°C. Enzyme activities were measured with veratryl alcohol and manganese sulfate as substrates, and the stability of the DyP enzyme at different temperatures was evaluated via relative enzyme activity analysis (Li et al., 2012). The optimum pH and the effects of pH on the stability of DyP were measured separately at 25°C in glycine-HCl buffer (pH 2.0–3.0), citrate buffer (pH 5.0–6.0), phosphate buffer (pH 6.0–8.0), Tris-HCl buffer (pH 8.0–9.0) and glycine-NaOH buffer (pH 9.0–10.0) (Falade et al., 2019). The enzyme activities of DyP were also determined in the presence of 1 mM metal salts (Mg^2+^, Ag^2+^, Cu^2+^, Zn^2+^, Fe^2+^, Co^2+^, Ba^2+^, Ca^2+^, Al^3+^, and Pb^2+^) and different chemical agents (EDTA, SDS, and Tween-80) (Falade et al., 2019).

### *DyP* Gene Knockout and Complementation in C2

The *DyP*gene was amplified by using the genome of *Arthrobacter* sp. C2 as the template. The *DyP* gene and the pUC57 vector were ligated to prepare the recombinant plasmid pUC57-*DyP*. The *Amp* gene was amplified using the pUC18 plasmid as the template and cloned to prepare pUC57-*Amp*. The recombinant pUC57-*DyP1-Amp*-*DyP2* plasmid was obtained by digesting (using *Cla*I and *Eco*NI) and ligating the pUC57-*DyP* and pUC57-*Amp* plasmids, which were selected by ampicillin resistance. The restriction endonucleases *Bam*HI and *Eco*RI were used to prepare the linear fragments of *DyP1-Amp*-*DyP2*. Then, the linear fragments were introduced into C2 by electroporation to induce homologous recombination (Zang et al., 2020).

The *DyP* gene sequence amplified by PCR was digested using *Bam*HI*/Eco*RI and ligated into *Bam*HI*/Eco*RI-digested pSET152. The pSET152-*DyP* plasmid was transformed into the mutant strain C2Δ*DyP*, yielding the complemented strain C2Δ*DyP*[pSET152- *DyP*] (Zang et al., 2020).

The abilities of the mutant and complemented strains to degrade lignin were determined by monitoring substrate degradation, cell growth and enzyme activities. The strains were inoculated with a 2% inoculum size (OD_600 nm_ = 1) in 100 mL of MSM containing 3 g/L sodium lignin sulfonate and incubated for 9 days at 15°C. The degradation rate, bacterial growth and enzyme activities were measured at 1-day intervals as described by Jiang et al. (2019).

### Comparative Transcriptomic Analysis of C2 and C2Δ*DyP*

Strains C2 and C2Δ*DyP* were cultured on lignin or glucose medium at 15°C for 72 h. All samples were pelleted by centrifugation at 8000 rpm and 4°C for 1 min followed by washing with diethyl pyrocarbonate-treated water. The harvested samples were immediately frozen in liquid nitrogen and stored at –80°C (Hou et al., 2021). Transcriptome sequencing was performed on the PacBio Sequel platform at Shanghai Personal Biotechnology Co., Ltd. All experiments were conducted in triplicate.

### Data Availability

The genome sequence of *Arthrobacter* sp. C2 has been deposited in the NCBI database under the accession number CP042428.

## Results

### Genomic Property Analysis

The complete genome of *Arthrobacter* sp. C2 was sequenced by using the Illumina MiSeq and PacBio platforms. The results showed that the chromosomal genome length was 4,555,954 bp, with a GC content of 66.23%. A total of 4159 open reading frames (ORFs) were identified, and the total length of the ORFs was 3,887,034 bp, with an average GC content of 67.06%. ncRNA prediction showed that there were 56 tRNAs and 16 rRNAs in the genome. The existence of a large number of tRNAs in the C2 genome is conducive to protein synthesis and growth (Talukdar et al., 2016). The tRNA content in bacteria is directly proportional to the growth rate because the growth of bacteria depends on protein synthesis. tRNA plays a central role in protein biosynthesis, which can promote protein production by regulating mRNA translation (Rocha, 2004). The prediction of GIs showed that there were 6 GIs in the C2 genome.

### Genetic Basis of Lignin Degradation in *Arthrobacter* sp. C2

**FIGURE 1:**
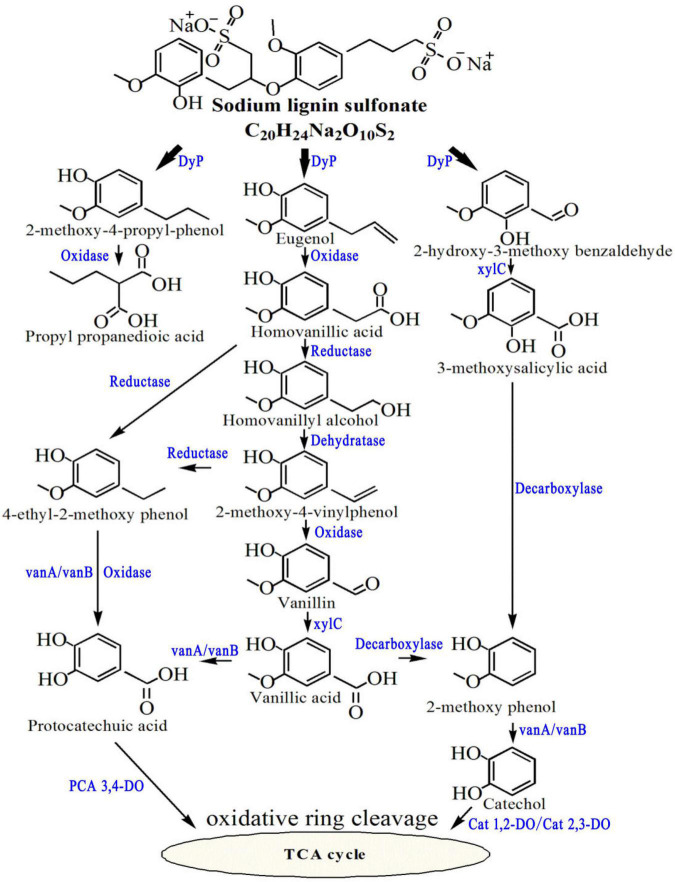
Proposed pathways for the metabolism of sodium lignin sulfonate by psychrotrophic *Arthrobacter* sp. C2. Part of the figure is cited from the author’s previous article (Jiang et al., 2019).

The degradation ability of C2 toward lignin is attributed to enzymes related to lignin degradation in the genome, including DyP, polyphenol oxidase, manganese superoxide dismutase, benzaldehyde dehydrogenase, decarboxylase, vanillate/3-*O*-methylgallate *O*-demethylase, vanillate *O*-demethylase oxygenase, catechol 1,2-dioxygenase, catechol 2,3-dioxygenase and protocatechol 3,4-dioxygenase (Table 1). In the author’s previous study, the possible pathways of lignin degradation by the psychrotrophic bacterium C2 were proposed, including oxidation, dehydrogenation, decarboxylation, demethylation and ring-opening reactions, which could be supported by the discovery of these enzymes (Figure 1).

**TABLE 1:** Comparison of the number of enzymes for lignin degradation in the genome of the psychrotrophic *Arthrobacter* sp. C2 strain and the other 4 lignin-degrading bacteria.

Enzymes	*Arthrobacter* sp. C2	*Rhodococcus jostii* RHA1	*Raoultella ornithinolytica* S12	*Sphingobium* sp. SYK-6	Halomonas sp. KO116
**Lignin-degrading enzymes**					
Polyphenol oxidase	1	0	2	0	0
DyP (dye-decolorizing peroxidase)	1	1	1	0	0
Superoxide dismutase [Mn]	2	0	1	1	1
**Lignin-degrading auxiliary enzymes**					
Glyoxalase	5	7	2	2	1
Benzaldehyde dehydrogenase	1	1	0	0	1
Decarboxylase	15	15	20	7	14
Dehydratase	27	61	28	23	25
Hydroxylase	11	33	15	21	16
Vanillate/3-*O*-methylgallate *O*-demethylase	1	0	0	0	0
Vanillate *O*-demethylase oxygenase	2	0	0	0	0
**Ring oxidation and ring cleavage**					
FAD-dependent oxidoreductase	9	21	7	5	10
Phenol 2-monooxygenase	3	0	0	0	1
Catechol 1,2-dioxygenase	1	3	1	0	3
Catechol 2,3-dioxygenase	2	3	0	0	0
4-Hydroxybenzoate 3-monooxygenase	1	1	1	1	2
Protocatechuate 3,4-dioxygenase	4	11	3	1	7
4-Carboxymuconolactone decarboxylase	1	1	1	2	0

### Low-Temperature Adaptability of Strain C2

The psychrotrophic strain C2 contains proteins and regulatory factors related to low-temperature adaptation (Supplementary Table 3). The proteins and factors related to the cold shock response include three cold shock proteins, four translation initiation factors, two transcription elongation factors, one ribosome-binding factor, and one polyribonucleotide transferase. These proteins and factors are related to the cold stress response and regulation (Boscari et al., 2004), including protein conformation, membrane adaptation and antioxidant stress. The presence of these enzymes contributes to the adaptation of the psychrotrophic strain C2 to a low-temperature environment.

### Detection of mRNA Expression of the Key Lignin-Degrading Gene *DyP* by RT–qPCR

Genomic sequence analysis indicated the presence of the *DyP* gene (C2_GM001845) in C2. The mRNA expression of the *DyP* gene was detected at different culture times (1, 2, 3, 4, 5, 6, 7, 8, and 9 days). The results showed that the expression level of *DyP* first increased, peaking at 7 days, and then decreased (Supplementary Figure 1). This trend is consistent with the generation of the first-step product during the degradation of lignin by the psychrotrophic strain C2. The first-step products 2-methoxy-4-propylphenol, eugenol and 2-hydroxy-3-methoxybenzaldehyde were detected at 1, 3, 5, and 7 days. At 9 days, only 2-methoxy-4-propylphenol was detected. The trend of the *DyP* expression level was consistent with that of enzyme activity (our previous study) (Jiang et al., 2019). According to the single-factor experiment results (Jiang et al., 2019), LiP activity and MnP activity showed a trend of first increasing and then decreasing, and the highest activities were observed at 7 days. In this study, the efficiency of sodium lignin sulfonate degradation by psychrotrophic strain C2 was highest at 7 days (Supplementary Table 4). The above analysis suggested that the *DyP* gene may be involved in lignin degradation by the psychrotrophic strain C2.

### Purification and Functional Analysis of DyP

**FIGURE 2:**
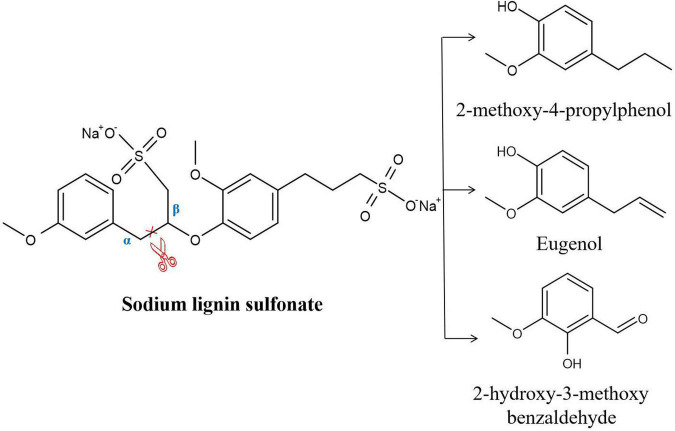
Sodium lignin sulfonate degradation products generated by purified DyP.

Recombinant DyP was expressed and purified. Analysis in an SDS–PAGE gel showed that the molecular weight of DyP was close to 42 kDa, which was consistent with the theoretical molecular mass of 44 kDa. The concentration of the purified DyP protein measured by the Bradford method was 137 μg/mL. The purified DyP was incubated with L-MSM, and the resulting enzyme activity and decolorization rate were measured. The results showed DyP activities of 16.8 U/L and 37.0 U/L against veratryl alcohol and manganese sulfate, respectively. The decolorization rate of lignin determined by UV spectrophotometry at 456 nm was 43.2%, indicating that DyP had the ability to degrade lignin. The degradation products were identified and analyzed by GC–MS (Supplementary Figure 2). The results indicated that eight products were detected in the treated samples, namely, 2-hydroxypropionic acid, 1,2-butanediol, phenol, benzoic acid, 4-hydroxybenzoic acid, 2-methoxy-4-propyl-phenol, eugenol and 2-hydroxy-3-methoxybenzaldehyde (Supplementary Table 3 and Supplementary Figure 2). Among these products, 4-hydroxypropionic acid, 1,2-butanediol, phenol, benzoic acid and 4-hydroxybenzoic acid were also detected in the control treatment. There was no direct evidence that these substances were hydrolyzates of lignin. Thus, it was speculated that the substances detected in the control group in this study may be derived from the lignin manufacturing process (Wang et al., 2013). In addition, 2-hydroxy-3-methoxybenzaldehyde, 2-methoxy-4-propyl-phenol and eugenol were found in only the DyP treatment group. These three products were also detected in the analysis of the sodium lignin sulfonate degradation pathway in the author’s previous study (Jiang et al., 2019). It was inferred that these substances may be the product of sodium lignin sulfonate degradation catalyzed by DyP. The reaction process is shown in Figure 2, and it can be seen that the cleavage of the Cα-Cβ bond was catalyzed by DyP.

### Characteristics of DyP

The relative enzyme activities of DyP were measured in the temperature range of 5–60°C. The results showed that DyP exhibited optimal enzyme activity at 25°C (Supplementary Figure 3a). Vieille and Zeikus (2001) pointed out that the optimal temperature range for cold-adapted enzyme activity was 5–25°C. The relative enzyme activity of DyP exceeded 90% at 20–30°C, and the highest enzyme activity was observed at 20–25°C, which was consistent with the optimal temperature range for cold-adapted enzyme activity. When the temperature exceeded 55°C, the enzyme activity of DyP decreased greatly. DyP showed fair stability over the range of 5–40°C, and its relative activity was higher than 90% (Supplementary Figure 3b). The stability decreased as the temperature increased. When the temperature reached 50°C, the relative enzyme activity dropped to approximately 50%. This finding indicates that DyP showed better thermal stability in a lower temperature range (5–40°C), which was conducive to the further development and industrial application of DyP (Falade et al., 2019). When the cold-adapted enzyme was used in the industrial production of valuable chemical products, the reaction could be quickly terminated by applying appropriate heating conditions (Struvay and Feller, 2012). The optimum pH, the effects of pH on the stability of DyP, and the effect of metal salts and different chemical agents on the enzyme activities of DyP were determined. The results indicated that the optimal activity of DyP occurred at pH 3.0 with veratryl alcohol as the mediator, while maximal enzyme activity was observed at pH 4.5 when manganese sulfate was used as the mediator (Supplementary Figure 3c). The relative enzyme activity of DyP was promoted by Mg^2+^, Cu^2+^, Zn^2+^, Fe^2+^, and Co^2+^, and the activity-promoting effect of Zn^2+^ was the strongest, resulting in a relative enzyme activity of 126.86% when veratryl alcohol was used as the mediator. When manganese sulfate was used as the mediator, Cu^2+^ showed the strongest activity-promoting effect, with the relative enzyme activity reaching 121.50%. Ag^2+^ and Ca^2+^ exerted no obvious effects on the relative enzyme activity of DyP. However, the relative enzyme activity of DyP was strongly inhibited by Ba^2+^, Al^3+^ and Pb^2+^ (Supplementary Figure 3e). The chelating agent EDTA had little effect on enzyme activity. The surfactant SDS had a strong inhibitory effect, but Tween 80 exerted a certain stimulatory effect on the activity of DyP (Supplementary Figure 3e).

### *DyP* Is Involved in the Degradation of Lignin by Strain C2

**FIGURE 3:**
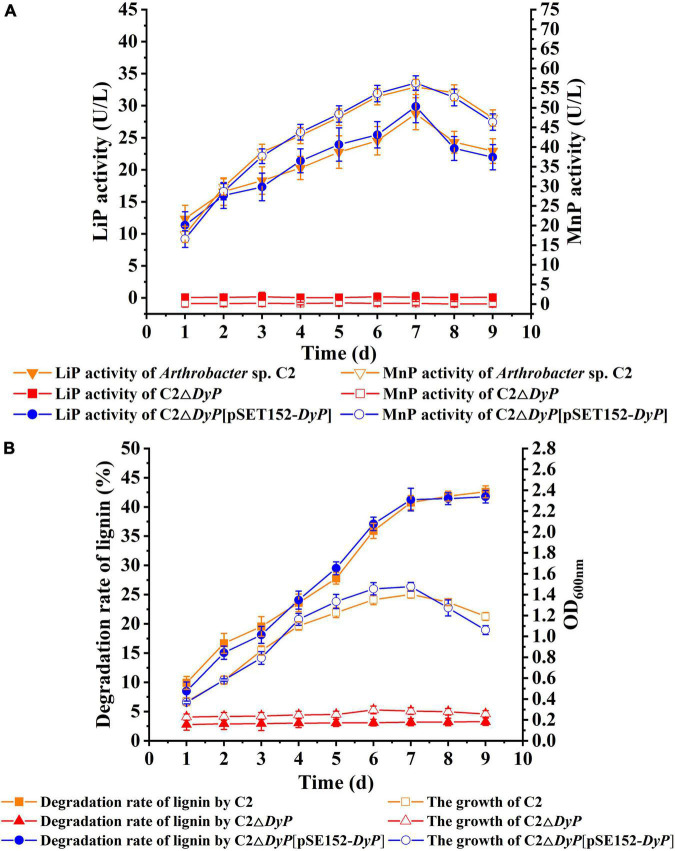
Functional verification of the role of the *DyP* gene in lignin degradation. **(A)** LiP and MnP enzyme activities of wild-type C2, C2Δ*DyP*, and C2Δ*DyP*[pSET152-*DyP*]; **(B)** growth and lignin degradation activity of wild-type C2, C2Δ*DyP*, and C2Δ*DyP*[pSET152-*DyP*].

To verify the function of the *DyP* gene in lignin degradation, the *DyP* deletion mutant strain C2Δ*DyP* and the complemented strain C2Δ*DyP*[pSET152-*DyP*] were constructed. The lignin degradation rates and the growth of C2Δ*DyP*, C2Δ*DyP*[pSET152-*DyP*] and the wild-type C2 strain were determined after incubation for 9 d in L-MSM (Figure 3). The results indicated that the growth of the wild-type C2 strain was better than that of the mutant strain C2Δ*DyP*. The rate of sodium lignin sulfonate degradation by C2 showed an increase with incubation time and reached 40.7% at 7 days, while the rate of degradation by C2Δ*DyP* was maintained at a low level at approximately 3%. At 7 days, the LiP activity and MnP activity of the wild-type C2 strain peaked at 28.4 U/L and 54.7 U/L, respectively. The LiP activity and MnP activity of C2Δ*DyP* were lower than 0.2 U/L and 0.3 U/L, respectively. The degradation rate, growth and enzyme activity of C2Δ*DyP*[pSET152-*DyP*] were similar to those of the wild-type C2 strain, indicating that supplementation with the pSET152-*DyP* plasmid restored the degradation ability of the mutant strain (Figure 3). The above results proved that *DyP* was involved in the degradation of lignin in C2.

### Comparative Transcriptomic Analysis of C2Δ*DyP* and C2 Grown on Lignin and Glucose Medium

**FIGURE 4:**
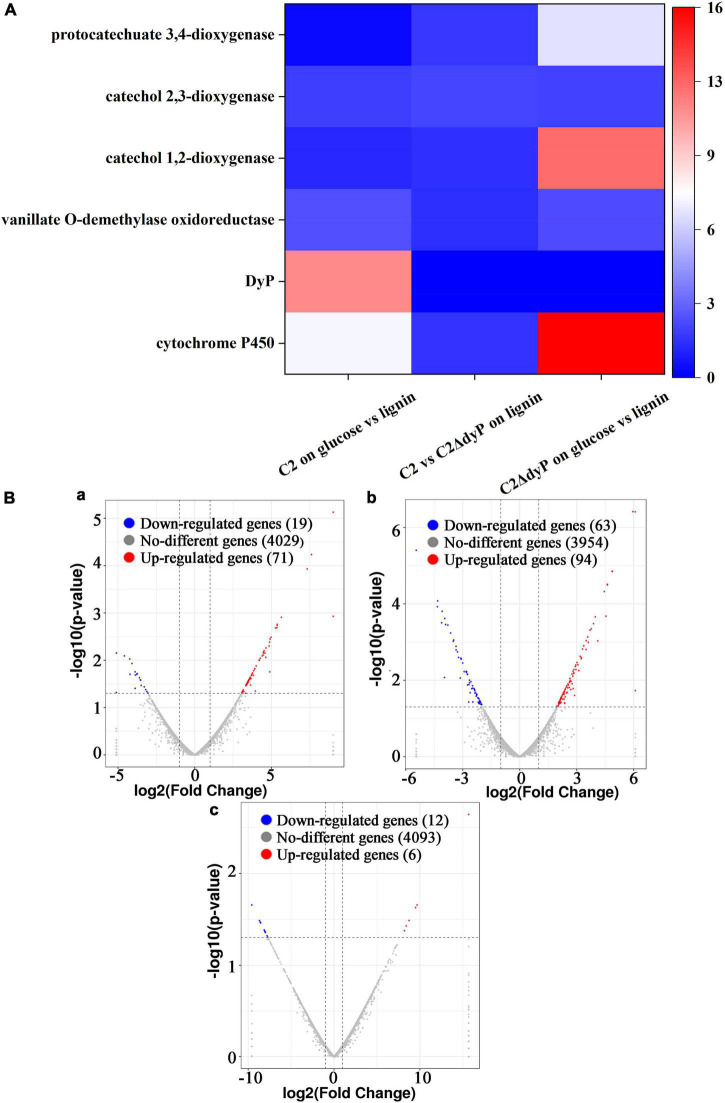
Differential expression analysis of lignin degradation genes. **(A)** Analysis of differentially expressed genes in three pairwise comparisons. Different colors indicate the fold change. **(B)** Volcano map of differentially expressed genes. **(Ba)** Wild-type strain C2 on glucose versus wild-type strain C2 on lignin. **(Bb)** Wild-type strain C2 on lignin versus mutant strain C2Δ*DyP* on lignin. **(Bc)** Mutant strain C2Δ*DyP* on glucose versus mutant strain C2Δ*DyP* on lignin.

To identify key genes in the pathways associated with lignin degradation (Jiang et al., 2019), three pairwise comparisons were performed, including C2 on glucose vs. lignin (wild-type strain C2 on glucose versus wild-type strain C2 on lignin), C2 vs. C2△*DyP* on lignin (wild-type strain C2 on lignin versus mutant strain C2Δ*DyP* on lignin), and C2△*DyP* on glucose vs. lignin (mutant strain C2Δ*DyP* on glucose versus mutant strain C2Δ*DyP* on lignin) (Figure 4). In the C2 on glucose vs. lignin group, 71 genes were upregulated, and 19 genes were downregulated (Supplementary Table 9). In this group, *DyP* was upregulated on lignin compared to glucose (*p* < 0.05) and was speculated to be the key gene that catalyzed the first reaction of lignin degradation (Ahmad et al., 2011). In the transcriptomic analysis of C2 on glucose vs. lignin, although some genes involved in the lignin degradation pathway of strain C2 were expressed, the difference was not significant. For example, the expression of the genes encoding the lignin-degrading enzymes aldehyde dehydrogenase (1.39-fold), vanillate *O*-demethylase oxidoreductase (2.51-fold), catechol 1,2-dioxygenase (1.25-fold) and catechol 2,3-dioxygenase (1.93-fold) increased, the expression of the protocatechuate 3,4-dioxygenase gene decreased, and the expression of other lignin-degrading enzyme genes showed no differential expression. The increased expression the above genes demonstrated that they were induced by lignin, and genes that showed no change may be constitutively expressed or were not differentially expressed due to the lack of induction of intermediate degradation products (An et al., 2020). The sites and modes of action of enzymes encoded by the above genes were consistent with the formation of 2-hydroxy-3-methoxybenzaldehyde, eugenol, 2-methoxy-4-propyl-phenol, 2-methoxy phenol, and vanillin in the degradation pathway (Jiang et al., 2019), suggesting that these genes participated in the biodegradation of lignin. Surprisingly, the cytochrome P450 gene was not present in the author’s previously predicted pathway, but it was significantly upregulated (*p* < 0.05). The upregulated expression of cytochrome P450 indicated that it may be correlated with the degradation of lignin in C2.

For the C2 vs. C2△*DyP* on lignin group, 94 upregulated genes and 63 downregulated genes were found in the *DyP* gene deletion mutant strain C2Δ*DyP* compared with the wild-type strain on the medium with lignin as the substrate (Supplementary Table 10). In the above study, the growth of the wild-type strain C2 was better than that of the mutant strain C2Δ*DyP*, and the lignin degradation rate, OD_600nm_, and maximum LiP activity and MnP activity of C2 reached 40.7%, 1.35, 28.4 U/L and 54.7 U/L, respectively. The degradation rate, OD_600nm_, LiP activity and MnP activity of the mutant strain C2Δ*DyP* were approximately 3%, 0.3, and 0.2 U/L and 0.3 U/L, respectively. Although deletion of the *DyP* gene had detrimental effects on the physiology of strain C2, the deletion strain could still metabolize substrates for growth, implying that there may be other genes responsible for the degradation of lignin. Based on the transcriptomic analysis, *DyP* was expressed in the wild-type strain but not in the mutant strain C2Δ*DyP*, and the transcriptional level of some other genes correlated with lignin degradation, such as genes encoding vanillate *O*-demethylase oxidoreductase (1.45-fold), catechol 1,2-dioxygenase (1.48-fold), catechol 2,3-dioxygenase (2.17-fold), and protocatechuate 3,4-dioxygenase (1.76-fold), increased. These enzymes catalyze the formation of the intermediate products of lignin degradation (Solyanikova et al., 2015).

Interestingly, when analyzing the differentially expressed genes of strain C2Δ*DyP* grown on lignin and on glucose (C2△*DyP* on glucose vs. lignin), compared with C2 vs. C2△*DyP* on lignin, the gene encoding cytochrome P450 was found to be significantly upregulated (15.67-fold) (*p* < 0.05). The induction of expression by lignin further confirmed the degradation-related role of P450 in strain C2Δ*DyP*. In addition, the fold upregulation of genes encoding vanillate *O*-demethylase oxidoreductase (2.39-fold), catechol 1,2-dioxygenase (12.37-fold), and protocatechuate 3,4-dioxygenase (6.94-fold) was enhanced, except for catechol 2,3-dioxygenase (2.02-fold) in group C2△*DyP* on glucose vs. lignin (Supplementary Table 11). These results further revealed that genetic compensation of strain C2Δ*DyP* allowed it to grow on lignin medium.

## Discussion

### Lignin Degradation Ability of C2

The psychrotrophic lignin-degrading *Arthrobacter* sp. C2 strain isolated in the author’s previous study showed a maximum degradation rate of 40% under optimal culture conditions of an initial pH of 6.74, temperature of 14.9°C, incubation time of 6.87 days, and inoculum size of 2.24% (Jiang et al., 2019). To further confirm the degradation ability of C2, its genome was analyzed. Genomic analysis showed that C2 contained unique genes related to lignin degradation, which was consistent with the lignin degradation pathway that we previously deduced. First, the Cα-Cβ bond of sodium lignin sulfonate was disrupted, resulting in the formation of 2-methoxy-4-propylphenol, eugenol and 2-hydroxy-3-methoxybenzaldehyde. A relevant study has shown that *DyP*B from *Rhodococcus jostii* RHA1 can catalyze the cleavage of the Cα-Cβ bond between adjacent benzene rings in lignin molecules and reduce the molecular weight of lignin (Ahmad et al., 2011). Therefore, it was inferred that the cleavage of the Cα-Cβ bond of sodium lignin sulfonate may be catalyzed by *DyP* in strain C2. The other enzymes found in the psychrotrophic bacterium C2 may be involved in the metabolism of lignin degradation intermediates. Among these enzymes, benzaldehyde dehydrogenase may catalyze the transformation of vanillin and 2-hydroxy-3-methoxybenzaldehyde to generate vanillic acid and 3-methoxysalicylic acid, respectively (Fleige et al., 2013). The vanillate *O*-demethylase oxygenase subunit and vanillate *O*-demethylase oxidoreductase may play a role in the conversion of vanillic acid to protocatechuic acid and 2-methoxyphenol to catechol (Priefert et al., 1997). Catechol 1,2-dioxygenase and catechol 2,3-dioxygenase may participate in the catechol ring-opening reaction. Protocatechuic acid 3,4-dioxygenase is involved in the protocatechuic acid ring-opening process (Solyanikova et al., 2015).

### Low-Temperature Viability of Strain C2

In cold areas, due to seasonal or diurnal temperature changes, the temperatures of industrial wastewater, groundwater and soil usually drop to approximately 15°C or lower. At this temperature, the activity of mesophilic degrading bacteria is greatly limited, but psychrotrophic bacteria have evolved low-temperature adaptation strategies to enable them to avoid the negative effects of low temperature (Margesin et al., 2013). In this work, unique genes associated with low-temperature adaptation endowed C2 with the ability to survive under low-temperature conditions. Three heat shock proteins were found in the psychrotrophic bacterium C2; these proteins play an important role in maintaining the normal conformation of proteins and other physiological functions. Heat shock proteins can be induced and increase the viability of microorganisms under a variety of adverse environmental stresses (Zhao P. et al., 2017). A fatty acid desaturase associated with membrane adaptation was also found in C2; this enzyme is critical for maintaining membrane permeability and fluidity at low temperatures (Dieser et al., 2010). In addition, isopentenyl diphosphate δ isomerase, geranylgeranyl pyrophosphate synthase, phytoene synthase and phytoene dehydrogenase are related to carotenoid synthesis. Carotenoids can improve the adaptability of strains to low temperature by stabilizing the cell membrane, maintaining proton permeability and promoting antioxidant stress (Dieser et al., 2010).

### Comparative Genomic Analysis

Some bacteria with lignin degradation capacity have been subjected to whole-genome sequencing; these bacteria include *R. jostii* RHA1 (McLeod et al., 2006), *R. ornithinolytica* S12 (Bao et al., 2015), *Sphingobium* sp. SYK-6 (Masai et al., 2012), and *Halomonas* sp. KO116 (O’Dell et al., 2015). Compared with the above four lignin-degrading bacteria, C2 has a higher GC content. Relevant reports have shown that the increase in GC content is related to the stronger environmental tolerance of a species (Talukdar et al., 2016). Therefore, the high GC content of C2 may confer stronger environmental adaptability. The C2 genome also contains a large number of tRNAs and can therefore be regarded as presenting the potential for high-level protein synthesis and a high growth rate (Rocha, 2004).

Genome analysis showed that the psychrotrophic strain C2 and the above four lignin-degrading bacteria contain a variety of lignin-degrading enzymes, lignin-degrading auxiliary enzymes and ring-opening enzymes (Table 1). Among them, DyP enzymes were found in strains C2, RHA1 and S12. It has been reported that only DyPB in RHA1 catalyzes the cleavage of the Cα-Cβ bond (Ahmad et al., 2011). In this study, the catalytic activity of DyP in strain C2 toward lignin was similar to that of the RHA1 strain. In the degradation pathway of C2, it was found that the Cα-Cβ bond of sodium lignin sulfonate was cleaved first. Strain C2 exhibited a strong lignin degradation ability at low temperature (15°C), suggesting that the DyP of the psychrotrophic strain C2 may play a catalytic role as a cold-adapted enzyme. Bioinformatics analysis showed that the tertiary structure of DyP in C2 exhibited a Greek key topology [Supplementary Figure 4A(a)], which conferred DyP with high flexibility and enhanced its adaptability to withstand low temperatures. The structure could enhance the binding ability of the enzyme to the substrate at low temperature (Höcker et al., 2001). However, the DyPs of RHA1 and S12 did not exhibit a Greek key topology [Supplementary Figures 4A(b,c)], and the function of DyP in strain S12 has not been confirmed. Although DyPB in RHA1 has been confirmed to degrade lignin by catalyzing the cleavage of the Cα-Cβ bond, the amino acid sequence similarity of DyPB and DyP in C2 is only 14.81%, indicating that they are different enzymes belonging to the same DyP superfamily (Błażewicz et al., 2006). In addition, vanillate/3-*O*-methylgallate *O*-demethylase and vanillate *O*-demethylase oxygenases were found in only the psychrotrophic strain C2 (Table 1). The demethylation activity of these two enzymes can convert vanillic acid to protocatechuic acid (Solyanikova et al., 2015). Vanillic acid and protocatechuic acid are intermediate products of lignin degradation (Wang et al., 2013). Under the action of protocatechuic acid 3,4-dioxygenase, protocatechuic acid undergoes a ring-opening reaction and enters the tricarboxylic acid cycle (Solyanikova et al., 2015). Compared with the four lignin-degrading bacteria, the psychrotrophic strain C2 contained abundant enzymes related to lignin degradation, which gave the strain a stronger lignin degradation ability.

The low-temperature adaptation enzymes in the genomes of the psychrotrophic strain C2 and 4 other lignin-degrading bacteria were analyzed (Supplementary Table 6). All five lignin-degrading bacteria contained enzymes related to the cold shock response and membrane adaptability. It is speculated that the other four strains may also exhibit low-temperature tolerance, but the roles of these enzymes need to be further confirmed. In addition, an isopentenyl diphosphate δ isomerase and geranylgeranyl pyrophosphate synthase associated with carotenoid synthesis were found only in C2.

To date, 28 *Arthrobacter* strains have been subjected to whole-genome sequencing. The genomic annotation results of 20 mesophilic strains and 8 low-temperature strains of *Arthrobacter* were compared (Tables 2, 3). The results showed that there were lignin-degrading enzymes, lignin-degrading auxiliary enzymes and ring-opening enzymes in all of these strains. It is worth mentioning that only 6 of the 28 strains had DyP, including three mesophilic strains and three low-temperature strains. By comparing the DyP tertiary structure of psychrotrophic strain C2 with those of the other three low-temperature *Arthrobacter* strains (Supplementary Figure 4), it was found that *Arthrobacter* sp. AQ5-05 and *Arthrobacter alpinus* R3.8 also had a Greek key topology. However, this structure was not found in the DyPs of three mesophilic strains of *Arthrobacter*, indicating that the Greek key topology may be associated with the low-temperature adaptation of enzymes (Höcker et al., 2001) but not in all low-temperature strains. In addition, compared with 20 mesophilic strains of *Arthrobacter*, the psychrotrophic strain C2 contained 8 unique enzymes, namely, polyphenol oxidase, benzaldehyde dehydrogenase, vanillate/3-*O*-methylgallate *O*-demethylase, vanillate *O*-demethylase, phenol-2-monooxygenase, catechol 2,3-dioxygenase, 4-hydroxybenzoate 3-monooxygenase and 4-carboxymuconolactone decarboxylase. Compared with 8 low-temperature *Arthrobacter* strains, the psychrotrophic strain C2 contained six specific enzymes, namely, polyphenol oxidase, benzaldehyde dehydrogenase, vanillate/methyl 3-*O*-demethylase, vanillate *O*-demethylase, phenol-2-monooxygenase and 4-hydroxybenzoate 3-monooxygenase. These findings improved the understanding of the functions of most of the above enzymes. In addition, polyphenol oxidase has the ability to degrade phenolic units in lignin (Kunito et al., 2009). Phenol-2-monooxygenase played a catalytic role in the conversion of phenol to catechol (Vardon et al., 2015). The function of 4-hydroxybenzoate 3-monooxygenase is to convert 4-hydroxybenzoic acid to protocatechuic acid (Shi et al., 2013). 4-Carboxymucolactone decarboxylase can catalyze the ring-opening reaction of protocatechuic acid (Eulberg et al., 1998).

**TABLE 2:** Comparison of enzymes for lignin degradation between psychrotrophic strain C2 and the other mesophilic *Arthrobacter* strains.

Enzymes	1	2	3	4	5	6	7	8	9	10	11	12	13	14	15	16	17	18	19	20	21
**Lignin-degrading enzymes**																					
Polyphenol oxidase	1	0	0	0	0	0	0	0	0	0	0	0	0	0	0	0	0	0	0	0	0
DyP(dye-decolorizing peroxidase)	1	0	0	0	0	0	0	1	0	0	1	0	0	0	0	0	0	0	0	2	0
Superoxide dismutase [Mn]	2	0	0	0	0	1	1	0	0	0	0	0	0	0	0	0	0	0	1	0	1
**Lignin-degrading auxiliary enzymes**																					
Glyoxalase	5	4	4	3	6	4	2	1	2	2	5	4	4	4	5	2	8	3	2	3	6
Benzaldehyde dehydrogenase	1	0	0	0	0	0	0	0	0	0	0	0	0	0	0	0	0	0	0	0	0
Decarboxylase	15	19	15	16	14	14	13	13	14	11	12	10	9	7	11	12	3	14	13	12	14
Dehydratase	27	25	27	25	31	25	29	28	28	24	26	23	16	19	25	24	26	28	27	19	30
Hydroxylase	11	15	12	9	10	11	10	7	5	17	8	6	7	3	6	17	9	9	13	5	10
Vanillate/3-*O*-methylgallate *O*-demethylase	1	0	0	0	0	0	0	0	0	0	0	0	0	0	0	0	0	0	0	0	0
Vanillate *O*-demethylase oxygenase	2	0	0	0	0	0	0	0	0	0	0	0	0	0	0	0	0	0	0	0	0
**Ring oxidation and ring cleavage**																					
FAD-dependent oxidoreductase	9	13	9	8	7	9	10	6	4	14	7	6	7	3	6	13	9	9	12	5	7
Phenol 2-monooxygenase	3	0	0	0	0	0	0	0	0	0	0	0	0	0	0	0	0	0	0	0	0
Catechol 1,2-dioxygenase	1	2	2	0	1	1	0	2	1	2	0	0	1	0	0	2	0	3	1	1	0
Catechol 2,3-dioxygenase	2	0	0	0	0	0	0	0	0	0	0	0	0	0	0	0	0	0	0	0	0
4-Hydroxybenzoate 3-monooxygenase	1	0	0	0	0	0	0	0	0	0	0	0	0	0	0	0	0	0	0	0	0
Protocatechuate 3,4-dioxygenase	4	6	5	3	3	4	0	5	2	6	4	3	4	0	3	7	2	3	5	3	3
4-Carboxymuconolactone decarboxylase	1	0	0	0	0	0	0	0	0	0	0	0	0	0	0	0	0	0	0	0	0

*1, Arthrobacter sp. C2; 2, Arthrobacter sp. 24S4-2; 3, Arthrobacter sp. YN; 4, Arthrobacter sp. QXT-31; 5, Arthrobacter sp. Rue61a; 6, Arthrobacter sp. FB24; 7, Arthrobacter sp. ZXY-2; 8, Arthrobacter sp. U41; 9, Arthrobacter sp. PGP41; 10, Arthrobacter sp. PAMC25564; 11, Arthrobacter sp. YC-RL1; 12, Arthrobacter sp. KBS0702; 13, Arthrobacter sp. UKPF54-2; 14, Arthrobacter sp. MN05-02; 15, Arthrobacter sp. LS16; 16, Arthrobacter crystallopoietes DSM 20117; 17, Arthrobacter dokdonellae DCT-5; 18, Arthrobacter phenanthrenivorans Sphe3; 19, Arthrobacter chlorophenolicus A6; 20, Arthrobacter arilaitensis Re117; 21, Arthrobacter aurescens TC1.*

**TABLE 3:** Comparison of enzymes for lignin degradation between the psychrotrophic strain C2 and the other low-temperature *Arthrobacter* strains.

Enzymes	1	2	3	4	5	6	7	8	9
**Lignin-degrading enzymes**									
Polyphenol oxidase	1	0	0	0	0	0	0	0	0
DyP (dye-decolorizing peroxidase)	1	1	0	1	0	0	1	0	0
Superoxide dismutase [Mn]	2	1	1	0	1	1	1	1	1
**Lignin-degrading auxiliary enzymes**									
Glyoxalase	5	5	6	4	1	3	4	5	7
Benzaldehyde dehydrogenase	1	0	0	0	0	0	0	0	0
Decarboxylase	15	11	18	9	11	18	9	8	11
Dehydratase	27	24	23	23	32	22	22	22	23
Hydroxylase	11	3	7	3	3	9	3	4	6
Vanillate/3-*O*-methylgallate *O*-demethylase	1	0	0	0	0	0	0	0	0
Vanillate *O*-demethylase oxygenase	2	0	0	0	0	0	0	0	0
**Ring oxidation and ring cleavage**									
FAD-dependent oxidoreductase	9	3	6	3	3	8	3	4	6
Phenol 2-monooxygenase	3	0	0	0	0	0	0	0	0
Catechol 1,2-dioxygenase	1	0	0	0	0	1	0	1	0
Catechol 2,3-dioxygenase	2	0	0	0	1	1	0	0	0
4-Hydroxybenzoate 3-monooxygenase	1	0	0	0	0	0	0	0	0
Protocatechuate 3,4-dioxygenase	4	0	1	0	2	4	0	1	1
4-Carboxymuconolactone decarboxylase	1	0	0	0	1	3	0	0	0

*1, Arthrobacter sp. C2; 2, Arthrobacter sp. PAMC 25486; 3, Arthrobacter sp.*

*ERGS1:01; 4, Arthrobacter sp. AQ5-05; 5, Arthrobacter sp. Hiyo4; 6, Arthrobacter sp. Hiyo8; 7, Arthrobacter alpinus R3.8; 8, Arthrobacter alpinus ERGS4:06; 9, Arthrobacter alpinus A3.*

The enzymes associated with low-temperature adaptation in the genomes of 20 mesophilic-temperature *Arthrobacter* strains and 8 low-temperature *Arthrobacter* strains were analyzed (Supplementary Tables 7, 8). It was found that both mesophilic and low-temperature *Arthrobacter* strains contained cold shock response and membrane adaptation-related enzymes. However, the comparison of strain C2 with other mesophilic *Arthrobacter* strains revealed the presence of translation initiation factor 5A, associated with the cold shock response, and an isopentenyl diphosphate δ isomerase was only found in the psychrotrophic strain C2. Moreover, fatty acid desaturase was found in the psychrotrophic strain C2, which is essential for maintaining membrane permeability and fluidity at low temperature (Dieser et al., 2010). Compared with other low-temperature *Arthrobacter* strains, the psychrotrophic strain C2 contains a unique isopentenyl diphosphate δ isomerase and geranyl pyrophosphate synthase.

### Genetic Compensation Mechanism of Strain C2

Comparative transcriptomic analysis showed that the induced transcriptional expression of *DyP* genes increased on lignin medium, and the mechanism of genetic compensation explained the survival strategy of the mutant strain C2Δ*DyP*. Cytochrome P450 has various roles in secondary metabolism and is thought to play a role in the biodegradation of xenobiotic compounds. According to the description by Brown and Chang (2014), cytochrome P450 can also breakdown lignin by hydroxylation or demethylation.

In this study, compared with C2 on glucose vs. lignin (wild-type strain C2 on glucose versus wild-type strain C2 on lignin), although the expression of the cytochrome P450 gene in C2 vs. C2Δ*DyP* on lignin (wild-type strain C2 on lignin versus mutant strain C2Δ*DyP* on lignin) was not significantly upregulated, the transcriptional level of the cytochrome P450 gene in the mutant strain was higher than that in the wild-type strain (3.03-fold), suggesting that it may be drawn into lignin utilization by strain C2 after *DyP* gene deletion. This phenomenon may be explained by the mechanism of genetic compensation, which is the ability of a living organism to maintain its viability and fitness in harsh natural conditions. The loss of one gene may be compensated by another with overlapping functions and expression patterns; therefore, the upregulation of related genes following gene knockout may be a direct consequence for genetic compensation (El-Brolosy and Stainier, 2017). Here, the increase in P450 expression in strain C2Δ*DyP* on lignin suggested that the ability of P450 was enhanced to compensate for the function of *DyP*. In summary, to survive on lignin medium after *DyP* gene deletion, strain C2 may induce a genetic compensation mechanism by increasing the expression of cytochrome P450 and related enzymes during lignin degradation.

## Conclusion

The psychrotrophic lignin-degrading *Arthrobacter* sp. C2 strain exhibited lignin degradation ability and low temperature adaptability. The cold-adapted DyP enzyme of C2 was involved in the catalytic cleavage of lignin Cα-Cβ bonds as a key lignin-degrading enzyme. Comparative transcriptomic analysis showed that the induced transcriptional expression of *DyP* genes increased on lignin medium, and the mechanism of genetic compensation explained the survival strategy of the mutant strain C2Δ*DyP*. These results provided important insights into the lignin metabolism mechanism of psychrotrophic bacteria, indicating the potential of C2 for application in lignin biodegradation and utilization in cold regions.

## Data Availability Statement

The data presented in this study are deposited in the NCBI database repository, accession number CP042428. We have verified that the data has been deposited by email reply on June 5, 2022 as follows: We have uploaded the genome data to the NCBI database and obtained the accession number (CP042428). We want to keep the data private until August 4, 2023. The proof that the data has been deposited in the form of a confirmation email from the repository. The datasets presented in this study can be found in online repositories.

## Author Contributions

CL, CJ, and HY designed the whole scheme of the study and conducted the experiments. CJ, HZ, ZL, and SS performed the experiments. HY, XS, YZ, LW, and HJ analyzed the data. CJ, HY, and CL wrote the manuscript. XS, YW, XZ, HW, and NH helped to revise the manuscript. All authors read and approved the final manuscript.

## Conflict of Interest

The authors declare that the research was conducted in the absence of any commercial or financial relationships that could be construed as a potential conflict of interest.

## Publisher’s Note

All claims expressed in this article are solely those of the authors and do not necessarily represent those of their affiliated organizations, or those of the publisher, the editors and the reviewers. Any product that may be evaluated in this article, or claim that may be made by its manufacturer, is not guaranteed or endorsed by the publisher.
