# Supplementary material for: Genome Functional Analysis of the Psychrotrophic Lignin-Degrading Bacterium Arthrobacter sp. C2 and the Role of DyP in Catalyzing Lignin Degradation
Source: Front Microbiol. 2022 Jul 13;13:921549. doi: 10.3389/fmicb.2022.921549 (PMC9327799; doi:10.3389/fmicb.2022.921549)
Supplement: Supplementary file 1 [file Data_Sheet_1.docx]

**Supplementary material**

**Genome functional analysis of the psychrotrophic lignin-degrading bacterium *Arthrobacter* sp. C2 and the role of DyP in catalyzing lignin degradation**

Cheng Jiang^1,2†^, Haohao Yan^1†^, Xiaohui Shen^2^, Yuting Zhang^1^, Yue Wang^1^,

Shanshan Sun^1^, Hanyi Jiang^1^, Hailian Zang^1^, Xinyue Zhao^1^, Ning Hou^1^, Ziwei Li^1^,

Liwen Wang^1^, Hanjun Wang^1^ and Chunyan Li^1*^

^1^ College of Resources and Environment, Northeast Agricultural University, Harbin, China,

^2^ College of Life Science and Resources and Environment, Yichun University, Yichun, China

Journal: Frontiers in Microbiology

* Corresponding author. Tel.: +86-0451-55190034

E-mail address: lcy032002@163.com (C. Y. Li)

^†^Cheng Jiang and HaohaoYan contributed equally to this work.

**Materials and methods**

*The RNA preparation process*

The total genomic DNA of C2 was extracted by using the Qiagen DNA extraction kit (Hilden, Germany) following the manufacturer’s instructions. PCRs were carried out in a 20 µL reaction volume containing 1 µL of cDNA, 2.5 µL of 10 × PCR buffer, 12.6 µL of ddH_2_O, 2 µL of dNTPs, 0.8 μL of each primer and 0.3 µL of Pfu Taq DNA Polymerase. The primers used to amplify the genes were 27F (AGAGTTTGATCCTGGCTCAG) and 1492R (GGTTACCTTGTTACGACTT). The conditions for PCR amplification of the gene were as follows: 5 min of denaturation at 95 ℃, followed by 30 cycles of denaturation at 95 ℃ for 50 s, annealing at 55 ℃ for 30 s, and extension at 72 ℃ for 2 min, and a final extension at 72 ℃ for 10 min; the samples were stored at 4 ℃. The amplified product was visualized in a 1.2% agarose gel, stained with ethidium bromide, and sequenced by Huada Technology Co., Ltd.

*The RT reaction, qPCR reaction and primer sequence*

To determine the expression of the*DyP* gene during lignin degradation, 16S rDNA was used as an internal reference gene, and the dynamic changes in the*DyP*gene were detected at the transcription level by RT–qPCR using the 2^-ΔΔCt^ method. Reactions were carried out in a 20 µL reaction volume. In addition, the primer sequences and the amplification reaction system are shown in Tables S1 and S2, respectively.

The reaction conditions were as follows: 95 ℃ for 3 min; 40 cycles of 95 ℃ for 5 s, 60 ℃ for 30 s, and 72 ℃ for 30 s. Each sample was replicated 3 times.

*The culture conditions for DyP production*

DyP production was performed in LB liquid medium containing 50 µg/ml kanamycin at 20 ℃ and 160 rpm.

*The purification process for DyP*

DyP expression was induced with 0.2 mM IPTG, and the protein was purified by Ni-NTA agarose (GE Healthcare, USA). The details of the procedure were described by Min et al.(Liu and Zhou, 2012; Min et al., 2019)

*The enzyme activity was determination*

The DyP activity was determined by monitoring the H_2_O_2_-dependent oxidation of veratryl alcohol (ε_310_ = 9300 mol^-1^ cm^-1^) and MnSO4 (ε_240_ = 8100 mol^-1^cm^-1^) at 0, 5, 10, 15, 20, 25, 30, 35, 40, 45, 50, 55 and 60 ℃. The reaction mixtures with veratryl alcohol as substrate contained veratryl alcohol (10 mmol L^-1^), 1 mL; tartaric acid buffer (0.1 mol L^-1^, pH 3), 1.5 mL; enzyme sample, 0.4 mL; and H_2_O_2_ (10 mmol L^-1^), 0.1 mL. The reaction mixtures with MnSO_4_ as substrate contained MnSO_4_ (15 mmol L^-1^), 0.5 mL; succinic acid buffer (0.05 mol L^-1^, pH 4.5), 2 mL; enzyme sample, 0.4 mL; and H_2_O_2_ (10 mmolL^-1^), 0.1 mL (Jiang et al., 2019).

*The construction process for theDyP deletion plasmid and the sequence ofDyP_1_-Amp-DyP_2_*

Before constructing the final recombinant *DyP_1_*-*Amp*-*DyP_2_* linear fragment, pUC57-*DyP* and pUC57-*Amp* were first constructed, pUC57-*DyP* was used as the backbone vector, and *Amp* was inserted between the*DyP* sequences of the target gene through enzyme digestion and enzyme linkage to obtain the recombinant *DyP_1_*-Amp-*DyP_2_* fragment.

The plasmids pUC57-*DyP* and pUC57-*Amp*were extracted, and the restriction enzymes *Cla*I and *Eco*NI were used to digest and link the above plasmids to obtain the recombinant plasmid pUC57-*DyP_1_*-Amp-*DyP_2._* Then, the recombinant plasmid pUC57-*DyP_1_*-*Amp*-*DyP_2_* was double digested with the restriction enzymes *Bam*HI and *Eco*RI to obtain the recombinant fragment *DyP_1_*-*Amp*-*DyP_2_*. The recombinant fragment was purified, and the fragment was recovered by 1% agarose gel electrophoresis.

The sequence of *DyP_1_*-*Amp*-*DyP_2_* is as follows:

TGCAGATTTCCGAATCCTGGATCAGGGCAGCCAGCGACGGGATGACCAGCGGCTTTGCCGTGCTGGAAAACCCCACGGACCATCCGGCCGCGCTCGTCAAGGTCGACTCGACCCTGGCCGGCAGCACCGAGCTGCACGACATGGAAGGCTCGGGCACCGCCATGAGCATGAAGGAGCTTGACGGTCCGCTGGAGATCCCCGCACATTCAACCATCGAGCTTGCCCCGGGTGGCAAACATGTGATGCTGATGGACCTGAAGCAGGAACTCAAGGTCGGTCAAAGCCGTGCCCTGAGACTCACCTTCGGCGACGGCAGCACCCGCGAGGTCCCCTTCGAGATCAAGAGCTTCACCGGGGCCAAGGAGTCCTACGCACCGGAAGCCGAGGAATCGGGCCACGCAGGTCCCGCGGAAGGGGACGGGCatgagtattcaacatttccgtgtcgcccttattcccttttttgcggcattttgccttcctgtttttgctcacccagaaacgctggtgaaagtaaaagatgctgaagatcagttgggtgcacgagtgggttacatcgaactggatctcaacagcggtaagatccttgagagttttcgccccgaagaacgttttccaatgatgagcacttttaaagttctgctatgtggcgcggtattatcccgtattgacgccgggcaagagcaactcggtcgccgcatacactattctcagaatgacttggttgagtactcaccagtcacagaaaagcatcttacggatggcatgacagtaagagaattatgcagtgctgccataaccatgagtgataacactgcggccaacttacttctgacaacgatcggaggaccgaaggagctaaccgcttttttgcacaacatgggggatcatgtaactcgccttgatcgttgggaaccggagctgaatgaagccataccaaacgacgagcgtgacaccacgatgcctgtagcaatggcaacaacgttgcgcaaactattaactggcgaactacttactctagcttcccggcaacaattaatagactggatggaggcggataaagttgcaggaccacttctgcgctcggcccttccggctggctggtttattgctgataaatctggagccggtgagcgtgggtctcgcggtatcattgcagcactggggccagatggtaagccctcccgtatcgtagttatctacacgacggggagtcaggcaactatggatgaacgaaatagacagatcgctgagataggtgcctcactgattaagcattggtaaTCCCCGGATACCGGTCCCGAGGTGCCGGCGGGTGGCGTGAGCCACCTTGGCACCTTGGTGCCGGGCAATCGGTGGGAGAATGGATCGGCGAGGAAGCTGAAAGGACATTGTGAACAAGTCACGGAACACGCAGGCCACGGCCACCAAGGTCGAGGCGTCGATGCCAGGCAAGAGGCTCTTGCTCATGCTGACCCTTCCCGCACTCGTGGTCGGGGTGCTGGTGATCATTGCGGCCAGCTATTTTGCCGGCACCGCCCAGGCCAGTGAGCTGGGGGACCCCGGGCCCTTCGTGCGGTGGGCGCTGCCTGCCGCCAAGGCACTTCACCACTCGTCGATGGCCATCACGATCGCCGCCCTGGTCTTCGCCGCAACCATCCTGCCGCGTTCCACCAAGCCCAAGCGCCCGGAACCGGGCAAGCACGACACCGACGGCGGCCAAACCCACCCCGCCTTTGCCCGCACCTTGAACCTTGCCGCCGCCTCCGGCATGGTCTGGACGGTGTCCGCGGCCGCGGTGCTGGTCTTCAACTTCTGGGAC

*The composition of MSM the glucose medium*

The mineral salt medium (MSM) consisted of 1.4 g of (NH_4_)_2_SO_4_, 0.5 g of MgSO_4_·7H_2_O, 2.0 g of K_2_HPO_4_, 0.3 g of CaCl_2_, 0.005 g of FeSO_4_·7H_2_O, 0.0016 g of MnSO_4_, 0.0017 g of ZnCl_2_, and 0.0017 g of CoCl_2_ (pH 7.0).

The glucose medium consisted of 3.0 g of glucose, 1.4 g of (NH_4_)_2_SO_4_, 0.5 g of MgSO_4_·7H_2_O, 2.0 g of K_2_HPO_4_, 0.3 g of CaCl_2_, 0.005 g of FeSO_4_·7H_2_O, 0.0016 g of MnSO_4_, 0.0017 g of ZnCl_2_, and 0.0017 g of CoCl_2_ (pH 7.0).


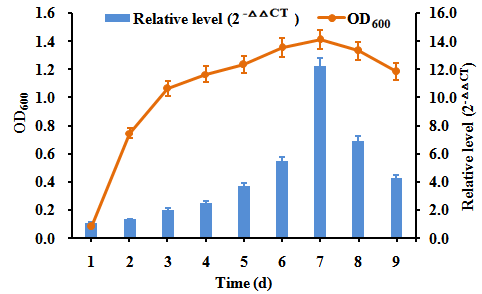


Fig. S1 The mRNA levels of the *DyP*genein the psychrotrophic*Arthrobacter* sp. C2 strain for lignin degradation


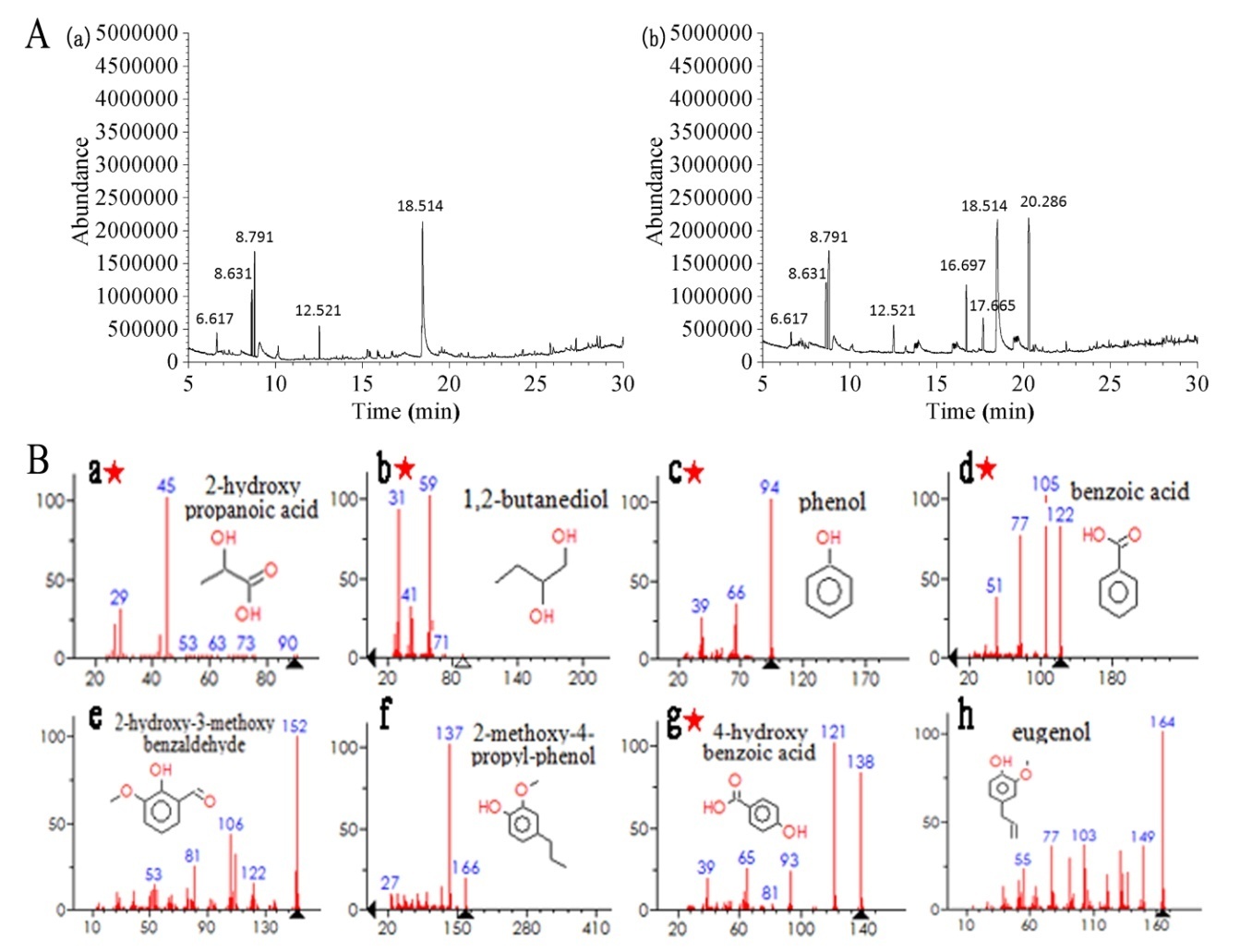


Fig. S2 GC–MS profile of sodium lignin sulfonate degradation products generated by purified DyP. A-a: Control group. A-b: Treatment group with purified DyP. B: GC–MS plots of sodium lignin sulfonate degradation products by purified DyP. The red stars indicate the compounds detected in the control sample.


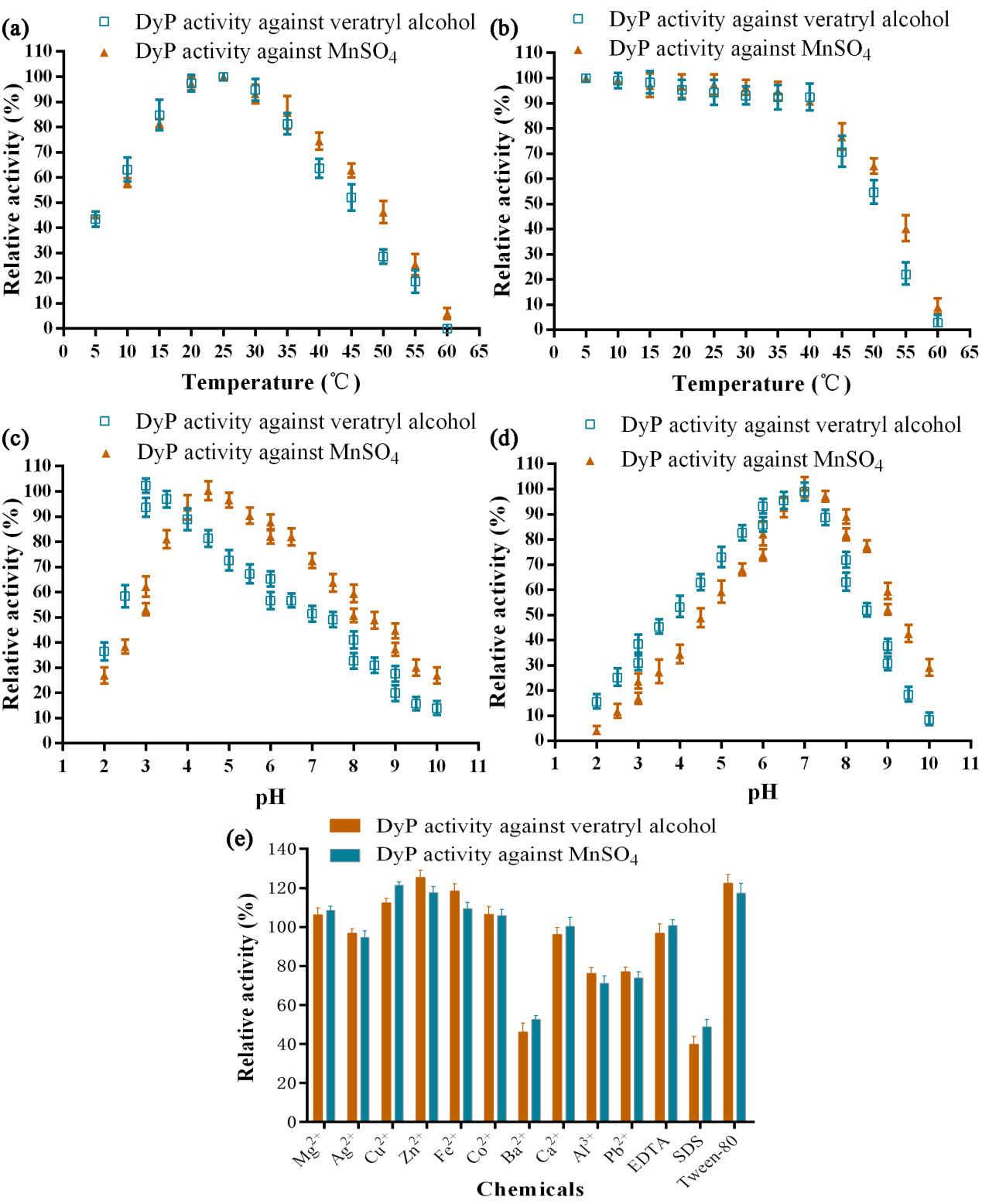


Fig. S3 Characteristics of DyP. (a) Effects of temperature on DyP activity. (b) Effects of temperature on DyP activity stability. (c) Effects of pH on DyP activity. (d) Effects of pH on DyP stability. (e) Effects of the different chemicals on DyP activity.


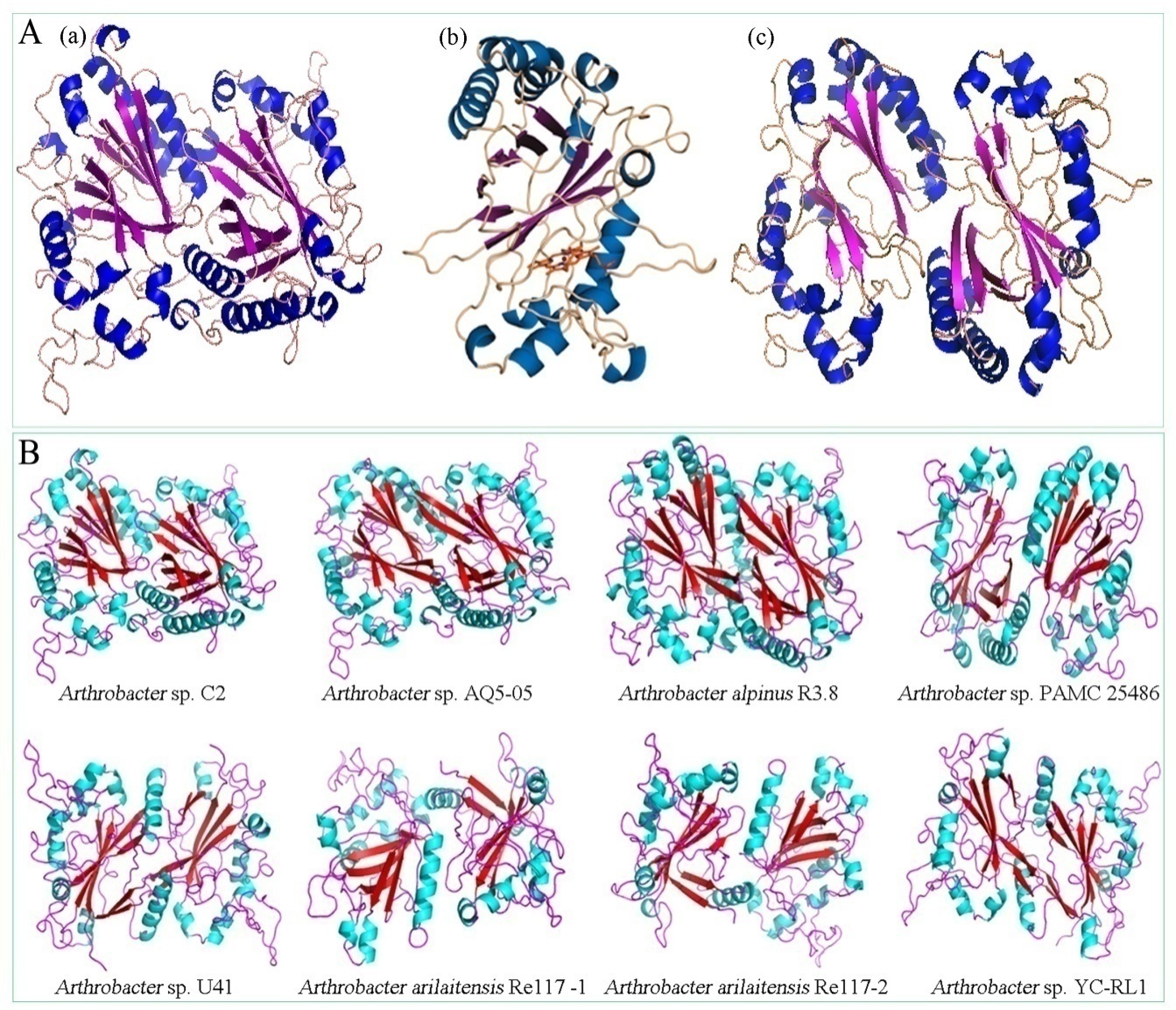


Fig. S4 Tertiary structures of DyP. A-a: DyP of *Arthrobacter* sp. C2. A-b: DyPB of*Rhodococcusjostii* RHA1. A-c: DyP of*Raoultellaornithinolytica* S12. B: Comparison of the three-dimensional structure of DyP between the psychrotrophic *Arthrobacter* sp. C2 strain and other *Arthrobacter* strains.


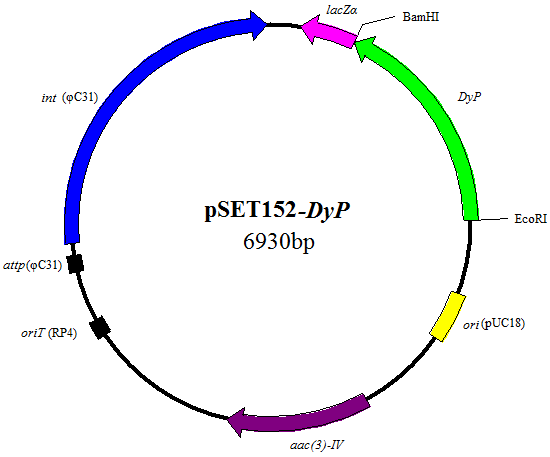


Fig. S5 The structure of pSET152-*DyP*


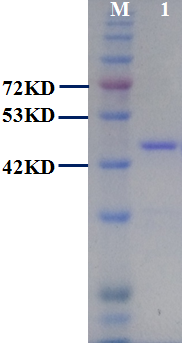


Fig. S6 SDS-PAGE analysis of DyP. M: Protein marker; 1: Purified recombinant protein DyP

Table S1 The primer sets used in RT-qPCR

| Target genes | Primer | Sequences (5＇→3＇) | Amplicon size (bp) |
| --- | --- | --- | --- |
| DyP | DyP-F | TTGCGGCCGCTCGATGCCATGCTTGATCG | 98 |
|  | DyP-R | CCCTCGAGCAGCACCAGTGAGG  TTCCAC |  |
| 16S | 16S-F | TCGTCTGTGAAATCCCGCAG | 171 |
|  | 16S-R | TTTCGCTCCTCAGCGTCAGT |  |

Table S2 RT-qPCR amplification reaction system

| Component | Volume |
| --- | --- |
| 2× Μniversal SYBR Green Fast qPCR Mix | 10μl |
| Template cDNA | 2μl |
| Forward Primer （10 μM） | 0.4μl |
| Reverse Primer（10 μM） | 0.4μl |
| Nμclease-free H_2_O | Up to 20.0μl |

Table S3 Proteins and regulatory factors related to low-temperature adaptation ofthe psychrotrophic*Arthrobacter* sp. C2 strain

| Enzymes | Gene_id |
| --- | --- |
| **Cold shock response** |  |
| Cold-shock protein | C2_GM001273、C2_GM002014、C2_GM003399 |
| Transcription elongation factor GreA | C2_GM003718、C2_GM003992 |
| Polyribonucleotide nucleotidyltransferase | C2_GM004096 |
| Ribosome-binding factor A | C2_GM003994 |
| Translation initiation factor IF-1 | C2_GM001513 |
| Translation initiation factor IF-2 | C2_GM003993 |
| Translation initiation factor IF-3 | C2_GM000015 |
| Translation initiation factor 5A (eIF-5A) | C2_GM000636 |
| **Membrane adaptations** |  |
| Fatty acid desaturase | C2_GM003643 |
| **Carotenoid biosynthesis** |  |
| Isopentenyldiphosphate δ isomerase | C2_GM002087 |
| Geranylgeranyl pyrophosphate synthase | C2_GM000180,C2_GM001618、C2_GM002309 |
| Phytoene synthase | C2_GM002310 |
| Phytoene desaturase | C2_GM002311 |
| Heat shock response |  |
| heat shock protein HtpX | C2_GM001606 |
| heat shock protein HspR | C2_GM001793 |
| ribosome-associated heat shock protein Hsp15 | C2_GM001984 |

Table S4 Efficiency of degradation of sodium lignin sulfonate by the psychrotrophic*Arthrobacter* sp. C2 strain

| Time (d) | Degradation rate (%) | Degradation efficiency (g/L/d) |
| --- | --- | --- |
| 1 | 10.2 | 0.061 |
| 2 | 16.8 | 0.079 |
| 3 | 21.0 | 0.094 |
| 4 | 23.8 | 0.121 |
| 5 | 29.8 | 0.132 |
| 6 | 34.4 | 0.188 |
| 7 | 42.0 | 0.225 |
| 8 | 43.3 | 0.041 |
| 9 | 44.2 | 0.028 |

Table S5 Identification of degradation products from sodium lignin sulfonate generated by purified DyP

| RT (min) | Control | Treatment | Compounds |
| --- | --- | --- | --- |
| 6.617 | + | + | 2-hydroxy propanoic acid |
| 8.631 | + | + | 1,2-butanediol |
| 8.791 | + | + | Phenol |
| 12.521 | + | + | benzoic acid |
| 16.697 | - | + | 2-hydroxy-3-methoxybenzaldehyde |
| 17.665 | - | + | 2-methoxy-4-propyl-phenol |
| 18.514 | + | + | 4-hydroxy benzoic acid |
| 20.286 | - | + | Eugenol |

Note: RT: retention time; +: present, -: absent.

Table S6 Comparison of enzymes related to low-temperature adaptation between the psychrotrophic*Arthrobacter* sp. C2 strain and the 4 other lignin-degrading bacteria

| Enzymes | *Arthrobacter* sp. C2 | *Rhodococcusjostii* RHA1 | *Raoultellaornithinolytica* S12 | *Sphingobium* sp. SYK-6 | Halomonas sp. KO116 |
| --- | --- | --- | --- | --- | --- |
| **Cold shock response** |  |  |  |  |  |
| Cold shock domain-containing protein | 0 | 3 | 1 | 0 | 1 |
| Cold-shock protein | 3 | 6 | 1 | 2 | 4 |
| Transcription elongation factor | 2 | 1 | 2 | 2 | 2 |
| Polyribonucleotide nucleotidyltransferase | 1 | 1 | 1 | 1 | 1 |
| Ribosome-binding factor A | 1 | 1 | 1 | 1 | 1 |
| Translation initiation factor IF-1 | 1 | 1 | 1 | 1 | 1 |
| Translation initiation factor IF-2 | 1 | 1 | 1 | 1 | 1 |
| Translation initiation factor IF-3 | 1 | 1 | 1 | 1 | 1 |
| Translation initiation factor 5A (eIF-5A) | 1 | 0 | 0 | 0 | 0 |
| **Membrane adaptations** |  |  |  |  |  |
| Fatty acid desaturase | 1 | 3 | 1 | 1 | 1 |
| **Carotenoid biosynthesis** |  |  |  |  |  |
| Isopentenyldiphosphate δ isomerase | 1 | 0 | 0 | 0 | 0 |
| Geranylgeranyl pyrophosphate synthase | 3 | 0 | 0 | 0 | 0 |
| Phytoene synthase | 1 | 1 | 0 | 1 | 0 |
| Phytoene desaturase | 1 | 1 | 0 | 1 | 0 |

Table S7 Comparison of enzymes related to low-temperature adaptation in the genomes of the psychrotrophic strain C2 and the 20 other mesophilic *Arthrobacter* strains

| Enzymes | 1 | 2 | 3 | 4 | 5 | 6 | 7 | 8 | 9 | 10 | 11 | 12 | 13 | 14 | 15 | 16 | 17 | 18 | 19 | 20 | 21 |
| --- | --- | --- | --- | --- | --- | --- | --- | --- | --- | --- | --- | --- | --- | --- | --- | --- | --- | --- | --- | --- | --- |
| **Cold shock response** |  |  |  |  |  |  |  |  |  |  |  |  |  |  |  |  |  |  |  |  |  |
| Cold shock domain-containing protein | 0 | 1 | 1 | 1 | 0 | 0 | 0 | 0 | 2 | 0 | 1 | 2 | 2 | 0 | 0 | 1 | 2 | 1 | 0 | 0 | 1 |
| Cold-shock protein | 3 | 5 | 3 | 4 | 4 | 5 | 4 | 7 | 3 | 5 | 4 | 3 | 3 | 3 | 5 | 5 | 3 | 4 | 6 | 3 | 4 |
| Transcription elongation factor GreA | 2 | 1 | 1 | 1 | 1 | 1 | 1 | 1 | 1 | 1 | 1 | 1 | 1 | 1 | 1 | 1 | 1 | 1 | 1 | 1 | 1 |
| Polyribonucleotide nucleotidyltransferase | 1 | 1 | 1 | 1 | 1 | 1 | 1 | 1 | 1 | 1 | 1 | 1 | 1 | 1 | 0 | 1 | 1 | 1 | 1 | 1 | 1 |
| Ribosome-binding factor A | 1 | 1 | 1 | 1 | 1 | 1 | 1 | 1 | 1 | 1 | 1 | 1 | 1 | 1 | 0 | 1 | 1 | 1 | 1 | 1 | 1 |
| Translation initiation factor IF-1 | 1 | 1 | 1 | 1 | 1 | 1 | 1 | 1 | 1 | 1 | 1 | 1 | 1 | 1 | 1 | 1 | 1 | 1 | 1 | 1 | 1 |
| Translation initiation factor IF-2 | 1 | 1 | 1 | 1 | 1 | 1 | 1 | 1 | 0 | 1 | 1 | 1 | 1 | 0 | 1 | 1 | 1 | 1 | 1 | 1 | 1 |
| Translation initiation factor IF-3 | 1 | 1 | 1 | 1 | 1 | 1 | 1 | 1 | 1 | 1 | 1 | 1 | 1 | 0 | 1 | 1 | 1 | 1 | 1 | 1 | 1 |
| Translation initiation factor 5A (eIF-5A) | 1 | 0 | 0 | 0 | 0 | 0 | 0 | 0 | 0 | 0 | 0 | 0 | 0 | 0 | 0 | 0 | 0 | 0 | 0 | 0 | 0 |
| **Membrane adaptations** |  |  |  |  |  |  |  |  |  |  |  |  |  |  |  |  |  |  |  |  |  |
| Fatty acid desaturase | 1 | 0 | 0 | 0 | 0 | 0 | 0 | 0 | 0 | 0 | 0 | 0 | 0 | 2 | 1 | 0 | 0 | 0 | 0 | 0 | 0 |
| **Carotenoid biosynthesis** |  |  |  |  |  |  |  |  |  |  |  |  |  |  |  |  |  |  |  |  |  |
| Isopentenyldiphosphate δ isomerase | 1 | 0 | 0 | 0 | 0 | 0 | 0 | 0 | 0 | 0 | 0 | 0 | 0 | 0 | 0 | 0 | 0 | 0 | 0 | 0 | 0 |
| Geranylgeranyl pyrophosphate synthase | 3 | 0 | 1 | 0 | 0 | 0 | 0 | 0 | 0 | 0 | 0 | 0 | 0 | 2 | 1 | 0 | 0 | 0 | 0 | 0 | 0 |
| Phytoene synthase | 1 | 0 | 1 | 0 | 1 | 0 | 1 | 0 | 0 | 0 | 1 | 0 | 0 | 0 | 0 | 0 | 1 | 0 | 0 | 1 | 1 |
| Phytoene desaturase | 1 | 0 | 1 | 0 | 1 | 0 | 1 | 0 | 0 | 0 | 1 | 0 | 0 | 0 | 0 | 0 | 1 | 0 | 0 | 1 | 1 |
| Lycopene cyclase domain-containing protein | 0 | 0 | 1 | 0 | 1 | 0 | 1 | 0 | 0 | 0 | 2 | 0 | 0 | 0 | 0 | 0 | 2 | 0 | 0 | 2 | 1 |

Note: 1, *Arthrobacter* sp. C2; 2; *Arthrobacter* sp. 24S4-2; 3, *Arthrobacter* sp. YN; 4, *Arthrobacter* sp. QXT-31; 5, *Arthrobacter* sp. Rue61a; 6, *Arthrobacter* sp. FB24; 7, *Arthrobacter* sp. ZXY-2; 8, *Arthrobacter* sp. U41; 9, *Arthrobacter* sp. PGP41; 10, *Arthrobacter* sp. PAMC25564; 11, *Arthrobacter* sp. YC-RL1; 12, *Arthrobacter* sp. KBS0702; 13, *Arthrobacter* sp. UKPF54-2; 14, *Arthrobacter* sp. MN05-02; 15, *Arthrobacter* sp. LS16; 16, *Arthrobactercrystallopoietes* DSM 20117; 17, *Arthrobacterdokdonellae* DCT-5; 18, *Arthrobacterphenanthrenivorans*Sphe3; 19, *Arthrobacterchlorophenolicus* A6; 20, *Arthrobacterarilaitensis* Re117; 21, *Arthrobacteraurescens* TC1

Table S8 Comparison of enzymes related to low-temperature adaptation in the genomes of the psychrotrophic strain C2 and the 8 other low-temperature *Arthrobacter* strains

| Enzymes | 1 | 2 | 3 | 4 | 5 | 6 | 7 | 8 | 9 |
| --- | --- | --- | --- | --- | --- | --- | --- | --- | --- |
| **Cold shock response** |  |  |  |  |  |  |  |  |  |
| Cold shock domain-containing protein | 0 | 3 | 2 | 1 | 0 | 0 | 1 | 1 | 1 |
| Cold-shock protein | 3 | 1 | 6 | 1 | 3 | 3 | 1 | 2 | 2 |
| Cold shock-like protein | 0 | 0 | 0 | 0 | 2 | 2 | 0 | 0 | 0 |
| Transcription elongation factor | 2 | 1 | 1 | 1 | 1 | 1 | 1 | 1 | 1 |
| Polyribonucleotide nucleotidyltransferase | 1 | 1 | 1 | 1 | 2 | 3 | 1 | 1 | 1 |
| Ribosome-binding factor A | 1 | 1 | 1 | 1 | 1 | 1 | 1 | 1 | 1 |
| Translation initiation factor IF-1 | 1 | 1 | 1 | 1 | 1 | 1 | 1 | 1 | 1 |
| Translation initiation factor IF-2 | 1 | 1 | 1 | 1 | 3 | 3 | 1 | 0 | 1 |
| Translation initiation factor IF-3 | 1 | 1 | 1 | 1 | 1 | 1 | 1 | 1 | 1 |
| Translation initiation factor 5A (eIF-5A) | 1 | 0 | 0 | 0 | 0 | 0 | 0 | 0 | 0 |
| **Membrane adaptations** |  |  |  |  |  |  |  |  |  |
| Fatty acid desaturase | 1 | 0 | 0 | 0 | 2 | 3 | 0 | 0 | 0 |
| **Carotenoid biosynthesis** |  |  |  |  |  |  |  |  |  |
| Isopentenyldiphosphate δ isomerase | 1 | 0 | 0 | 0 | 0 | 0 | 0 | 0 | 0 |
| Geranylgeranyl pyrophosphate synthase | 3 | 0 | 0 | 0 | 0 | 0 | 0 | 0 | 0 |
| Phytoene synthase | 1 | 1 | 1 | 1 | 0 | 0 | 1 | 1 | 1 |
| Phytoene desaturase | 1 | 1 | 1 | 1 | 0 | 0 | 1 | 0 | 1 |
| Lycopene cyclase domain-containing protein | 0 | 2 | 2 | 2 | 0 | 0 | 2 | 1 | 2 |

Note: 1, *Arthrobacter* sp. C2; 2, *Arthrobacter* sp. PAMC 25486; 3, *Arthrobacter* sp. ERGS1:01; 4, *Arthrobacter* sp. AQ5-05; 5, *Arthrobacter* sp. Hiyo4; 6, *Arthrobacter* sp. Hiyo8; 7, *Arthrobacter alpinus* R3.8; 8, *Arthrobacter alpinus* ERGS4:06; 9, *Arthrobacter alpinus* A3

Table S9 Gene expression in the group of C2 on glucose vs lignin

| **id** | **Base Mean** | **Fold Change(D/B)** | **log2 Fold**  **Change** | **pval** | **padj** | **Length** | **Description** | Level |
| --- | --- | --- | --- | --- | --- | --- | --- | --- |
| C2_GM003649 | 5503.49 | 520.52 | 9.02 | 0.000007 | 0.031 | 1254 | nitrite reductase (NAD(P)H) large subunit | up |
| C2_GM003651 | 12317.22 | 7.61 | 2.03 | 0.000059 | 0.121 | 1284 | cytochrome P450 for pimelic acid formation for biotin biosynthesis | up |
| C2_GM002435 | 1112.99 | 160.72 | 7.33 | 0.000117 | 0.161 | 1281 | cytochrome | up |
| C2_GM002436 | 51.76 | Inf | Inf | 0.001180 | 0.948 | 324 | ferredoxin | up |
| C2_GM002314 | 1833.35 | 50.23 | 5.65 | 0.001235 | 0.948 | 858 | prenyltransferase | up |
| C2_GM001378 | 20119.25 | 41.53 | 5.38 | 0.001745 | 0.948 | 615 | RNA polymerase sigma factor SigE | up |
| C2_GM000957 | 2588.72 | 41.42 | 5.37 | 0.001828 | 0.948 | 738 | chloramphenicol O-acetyltransferase | up |
| C2_GM003650 | 848.41 | 41.03 | 5.36 | 0.002048 | 0.948 | 324 | Rhodocoxin | up |
| C2_GM000890 | 6322.81 | 38.60 | 5.27 | 0.002071 | 0.948 | 561 | hypothetical protein ADIAG_02282 | up |
| C2_GM002315 | 3776.50 | 31.41 | 4.97 | 0.003261 | 1.000 | 1401 | FAD binding domain of DNA photolyase | up |
| C2_GM001377 | 6783.57 | 30.00 | 4.91 | 0.003556 | 1.000 | 879 | putative transmembrane transcriptional regulator (anti-sigma factor) | up |
| C2_GM002826 | 820.96 | 29.33 | 4.87 | 0.004122 | 1.000 | 963 | hydroxymethylglutaryl-CoA lyase | up |
| C2_GM002827 | 328.68 | 28.85 | 4.85 | 0.004997 | 1.000 | 990 | D-isomer specific 2-hydroxyacid dehydrogenase NAD-binding | up |
| C2_GM002825 | 1535.81 | 24.95 | 4.64 | 0.005491 | 1.000 | 1245 | CoA-transferase | up |
| C2_GM001655 | 6184.52 | 22.98 | 4.52 | 0.006269 | 1.000 | 636 | polysaccharide deacetylase | up |
| C2_GM001052 | 6654.40 | 22.28 | 4.48 | 0.006689 | 1.000 | 978 | membrane-bound transcriptional regulator LytR | up |
| C2_GM003648 | 1621.56 | 22.25 | 4.48 | 0.006952 | 1.000 | 918 | LysR family transcriptional regulator | up |
| C2_GM000888 | 398.02 | 23.79 | 4.57 | 0.007008 | 1.000 | 741 | ribonuclease HII | up |
| C2_GM002312 | 581.56 | 22.05 | 4.46 | 0.007694 | 1.000 | 402 | C50 carotenoid epsilon cyclase | up |
| C2_GM002227 | 870.95 | 21.07 | 4.40 | 0.008091 | 1.000 | 1308 | MFS transporter | up |
| C2_GM001905 | 153.63 | 24.76 | 4.63 | 0.008649 | 1.000 | 321 | hypothetical protein ADIAG_01898 | up |
| C2_GM000612 | 8167.30 | 19.25 | 4.27 | 0.009069 | 1.000 | 1734 | protein-export membrane protein SecD | up |
| C2_GM002824 | 5087.62 | 18.49 | 4.21 | 0.009918 | 1.000 | 1446 | gluconate permease | up |
| C2_GM000170 | 85574.81 | 18.29 | 4.19 | 0.010021 | 1.000 | 2727 | preprotein translocase subunit SecA | up |
| C2_GM002311 | 11012.91 | 16.05 | 4.00 | 0.013206 | 1.000 | 1623 | phytoene desaturase | up |
| C2_GM002352 | 6156.03 | 15.05 | 3.91 | 0.015133 | 1.000 | 777 | DeoR family transcriptional regulator | up |
| C2_GM000677 | 19286.99 | 14.99 | 3.91 | 0.015159 | 1.000 | 891 | hypothetical protein | up |
| C2_GM002924 | 4462.67 | 14.39 | 3.85 | 0.016641 | 1.000 | 1152 | neutral protease | up |
| C2_GM002313 | 42.24 | 29.69 | 4.89 | 0.017550 | 1.000 | 357 | C50 carotenoid epsilon cyclase | up |
| C2_GM002310 | 9257.53 | 13.62 | 3.77 | 0.018489 | 1.000 | 933 | squalene synthase HpnD | up |
| C2_GM003186 | 3549.11 | 13.30 | 3.73 | 0.019590 | 1.000 | 1500 | DNA-binding protein | up |
| C2_GM003562 | 264.27 | 14.16 | 3.82 | 0.020758 | 1.000 | 1065 | spermidine/putrescine ABC transporter ATP-binding protein | up |
| C2_GM002437 | 1384.56 | 13.04 | 3.70 | 0.020864 | 1.000 | 1263 | pyridine nucleotide-disulfide oxidoreductase | up |
| C2_GM002569 | 3055.79 | 12.88 | 3.69 | 0.020958 | 1.000 | 690 | succinyl-CoA--3-ketoacid-CoA transferase subunit A | up |
| C2_GM000742 | 12228.17 | 12.19 | 3.61 | 0.023091 | 1.000 | 948 | FKBP-type 22 kDa peptidyl-prolyl cis-trans isomerase | up |
| C2_GM002234 | 3158.51 | 12.21 | 3.61 | 0.023287 | 1.000 | 1101 | mandelate racemase | up |
| C2_GM002101 | 1963.19 | 12.25 | 3.61 | 0.023379 | 1.000 | 765 | hypothetical protein | up |
| C2_GM000613 | 395.07 | 12.67 | 3.66 | 0.024140 | 1.000 | 984 | protein-export membrane protein SecF | up |
| C2_GM002137 | 1988.23 | 11.90 | 3.57 | 0.024762 | 1.000 | 615 | Helix-turn-helix domain protein | up |
| C2_GM001364 | 781.07 | 12.10 | 3.60 | 0.024878 | 1.000 | 942 | adenine-specific DNA methylase | up |
| C2_GM002308 | 17913.42 | 11.66 | 3.54 | 0.025228 | 1.000 | 501 | MarR family transcriptional regulator | up |
| C2_GM002353 | 3500.18 | 11.65 | 3.54 | 0.025544 | 1.000 | 981 | fructose 1-phosphate kinase | up |
| C2_GM001845 | 4371.67 | 11.46 | 3.52 | 0.026351 | 1.000 | 1233 | dyp-type peroxidase family protein | up |
| C2_GM002873 | 2052.93 | 11.33 | 3.50 | 0.027277 | 1.000 | 333 | - | up |
| C2_GM001798 | 7042.20 | 11.20 | 3.49 | 0.027443 | 1.000 | 1749 | ATP-dependent RNA helicase | up |
| C2_GM003396 | 2797.09 | 11.10 | 3.47 | 0.028239 | 1.000 | 1470 | two-component sensor histidine kinase | up |
| C2_GM002309 | 14271.46 | 10.92 | 3.45 | 0.028756 | 1.000 | 912 | trans-hexaprenyltranstransferase | up |
| C2_GM003358 | 2846.67 | 10.86 | 3.44 | 0.029439 | 1.000 | 1038 | ABC transporter substrate-binding protein | up |
| C2_GM003790 | 706.13 | 11.12 | 3.47 | 0.029506 | 1.000 | 504 | aminoacyl-tRNA hydrolase | up |
| C2_GM000665 | 1173.69 | 10.90 | 3.45 | 0.029917 | 1.000 | 732 | hypothetical protein | up |
| C2_GM003791 | 7196.37 | 10.64 | 3.41 | 0.030357 | 1.000 | 1263 | cysteine desulfurase | up |
| C2_GM002226 | 4677.69 | 10.65 | 3.41 | 0.030416 | 1.000 | 840 | 1,6-dihydroxycyclohexa-2,4-diene-1-carboxylate dehydrogenase | up |
| C2_GM002136 | 1006.02 | 10.66 | 3.41 | 0.031415 | 1.000 | 1278 | hypothetical protein ADIAG_02145 | up |
| C2_GM002233 | 2349.91 | 10.51 | 3.39 | 0.031525 | 1.000 | 261 | muconolactone delta-isomerase | up |
| C2_GM002212 | 8175.60 | 10.32 | 3.37 | 0.032238 | 1.000 | 609 | FMN reductase | up |
| C2_GM002213 | 1265.72 | 10.45 | 3.38 | 0.032405 | 1.000 | 1665 | peptide ABC transporter oligopeptide-binding protein AppA | up |
| C2_GM000231 | 2742.63 | 10.30 | 3.36 | 0.032708 | 1.000 | 855 | M56 domain-containing peptidase | up |
| C2_GM002073 | 1374.35 | 10.35 | 3.37 | 0.032932 | 1.000 | 903 | hypothetical protein | up |
| C2_GM002828 | 105.33 | 12.50 | 3.64 | 0.033485 | 1.000 | 987 | glycerate kinase | up |
| C2_GM000402 | 19952.60 | 10.05 | 3.33 | 0.033873 | 1.000 | 789 | transcriptional regulator | up |
| C2_GM002570 | 1884.96 | 10.07 | 3.33 | 0.034402 | 1.000 | 633 | succinyl-CoA--3-ketoacid-CoA transferase subunit B | up |
| C2_GM003278 | 13285.87 | 8.99 | 3.17 | 0.042140 | 1.000 | 594 | transcriptional regulator | up |
| C2_GM003397 | 6239.70 | 8.74 | 3.13 | 0.044611 | 1.000 | 711 | DNA-binding response regulator | up |
| C2_GM000172 | 37.71 | 15.44 | 3.95 | 0.044653 | 1.000 | 792 | hypothetical protein | up |
| C2_GM003643 | 17316.70 | 8.70 | 3.12 | 0.044802 | 1.000 | 1095 | Stearoyl-CoA 9-desaturase | up |
| C2_GM001312 | 276.08 | 9.20 | 3.20 | 0.046106 | 1.000 | 807 | Mn2+/Zn2+ ABC transporter ATP-bindingprotein | up |
| C2_GM002571 | 2227.69 | 8.63 | 3.11 | 0.046189 | 1.000 | 1170 | acetyl-CoA acetyltransferase | up |
| C2_GM000327 | 3510.07 | 8.43 | 3.08 | 0.048017 | 1.000 | 1431 | glutamate synthase subunit beta | up |
| C2_GM003106 | 1353.87 | 8.46 | 3.08 | 0.048551 | 1.000 | 429 | CoA-binding domain-containing protein | up |
| C2_GM002354 | 4839.62 | 8.36 | 3.06 | 0.048689 | 1.000 | 2070 | PTS fructose transporter subunit IIABC | up |
| C2_GM002923 | 100.72 | 9.98 | 3.32 | 0.049831 | 1.000 | 318 | hypothetical protein | up |
| C2_GM002344 | 85.37 | 1.39 | 0.47 | 0.769788 | 1.000 | 1503 | aldehyde dehydrogenase (NAD) | increased |
| C2_GM002686 | 288.09 | 2.51 | 1.33 | 0.3778150 | 1.000 | 753 | Vanillate O-demethylase oxidoreductase | increased |
| C2_GM002232 | 16268.70 | 1.25 | 0.32 | 0.8221262 | 1.000 | 855 | catechol 1,2-dioxygenase | increased |
| C2_GM002345 | 254.54 | 1.93 | 0.95 | 0.5253341 | 1.000 | 831 | catechol 2,3-dioxygenase | increased |
| C2_GM001469 | 96.32 | 0.03 | -5.10 | 0.006994609 | 1 | 1134 | flagellar hook protein | down |
| C2_GM000043 | 231.33 | 0.04 | -4.58 | 0.008071945 | 1 | 1218 | ROK family protein | down |
| C2_GM001114 | 19292.26 | 0.05 | -4.24 | 0.009386339 | 1 | 261 | MULTISPECIES: membrane protein | down |
| C2_GM003235 | 14048.23 | 0.06 | -4.09 | 0.011682465 | 1 | 3249 | subtilisin-like serine protease | down |
| C2_GM000335 | 481.99 | 0.07 | -3.89 | 0.017559295 | 1 | 1383 | aspartate aminotransferase family protein | down |
| C2_GM002114 | 4549.82 | 0.07 | -3.75 | 0.019101198 | 1 | 294 | hypothetical protein | down |
| C2_GM001471 | 85.57 | 0.05 | -4.22 | 0.019740082 | 1 | 1317 | - | down |
| C2_GM003226 | 563.03 | 0.07 | -3.78 | 0.01989931 | 1 | 822 | MULTISPECIES: hypothetical protein | down |
| C2_GM001112 | 221.55 | 0.07 | -3.87 | 0.020517874 | 1 | 264 | hypothetical protein | down |
| C2_GM002423 | 5555.52 | 0.08 | -3.60 | 0.023355363 | 1 | 1293 | MFS transporter | down |
| C2_GM002424 | 1571.34 | 0.09 | -3.54 | 0.026026252 | 1 | 792 | short-chain dehydrogenase | down |
| C2_GM002425 | 2271.73 | 0.09 | -3.53 | 0.026173138 | 1 | 753 | 3-oxoacyl-ACP reductase | down |
| C2_GM003966 | 200.33 | 0.09 | -3.49 | 0.034003006 | 1 | 1005 | C4-dicarboxylate ABC transporter substrate-binding protein, partial | down |
| C2_GM002833 | 1927.27 | 0.10 | -3.29 | 0.036433408 | 1 | 660 | pterin-4-alpha-carbinolamine dehydratase | down |
| C2_GM003969 | 51.44 | 0.07 | -3.88 | 0.039241955 | 1 | 411 | universal stress protein UspA | down |
| C2_GM001111 | 1258.34 | 0.11 | -3.20 | 0.041733815 | 1 | 357 | hypothetical protein | down |
| C2_GM000703 | 1367.91 | 0.11 | -3.13 | 0.045754341 | 1 | 558 | hypothetical protein | down |
| C2_GM001475 | 19.36 | 0.03 | -5.11 | 0.047875903 | 1 | 615 | hypothetical protein | down |
| C2_GM001498 | 43961.53 | 0.12 | -3.06 | 0.048754117 | 1 | 372 | large-conductance mechanosensitive channel | down |

Table S10 Gene expression in the group of C2 vs C2*△dyp* on lignin

| **id** | **Base Mean** | **Fold Change(C/D)** | **log2 Fold**  **Change** | **pval** | **padj** | **Length** | **Description** | **Level** |
| --- | --- | --- | --- | --- | --- | --- | --- | --- |
| C2_GM000667 | 31128.59 | 61.91 | 5.95 | 3.85132E-07 | 0.0008 | 1845 | FAD-binding monooxygenase | up |
| C2_GM004090 | 1441.98 | 67.74 | 6.08 | 3.90185E-07 | 0.0008 | 372 | hypothetical protein | up |
| C2_GM003204 | 3130.82 | 29.25 | 4.87 | 1.4065E-05 | 0.0116 | 1791 | pyridine nucleotide-disulfide oxidoreductase | up |
| C2_GM003335 | 2589.58 | 29.37 | 4.88 | 1.41408E-05 | 0.0116 | 369 | - | up |
| C2_GM001096 | 3611.00 | 24.46 | 4.61 | 3.09138E-05 | 0.0187 | 1044 | glycosyl transferase family 1 | up |
| C2_GM004091 | 2930.52 | 24.40 | 4.61 | 3.19074E-05 | 0.0187 | 357 | hypothetical protein | up |
| C2_GM001097 | 17305.30 | 21.88 | 4.45 | 4.76874E-05 | 0.0245 | 2157 | amylo-alpha-1,6-glucosidase | up |
| C2_GM000074 | 143.53 | 23.21 | 4.54 | 0.00021013 | 0.0689 | 495 | - | up |
| C2_GM004089 | 2685.74 | 15.78 | 3.98 | 0.000217997 | 0.0689 | 294 | - | up |
| C2_GM004140 | 1097.15 | 14.73 | 3.88 | 0.000329239 | 0.0824 | 1224 | salicylate hydroxylase | up |
| C2_GM004088 | 1121.53 | 13.64 | 3.77 | 0.000451534 | 0.0977 | 225 | AlpA family transcriptional regulator | up |
| C2_GM003217 | 4595.64 | 12.98 | 3.70 | 0.000486747 | 0.1001 | 1167 | hypothetical protein | up |
| C2_GM003336 | 1857.40 | 11.93 | 3.58 | 0.000736522 | 0.1376 | 1527 | Tripeptidyl aminopeptidase precursor | up |
| C2_GM001129 | 2279.18 | 11.25 | 3.49 | 0.000922799 | 0.1505 | 492 | hypothetical protein | up |
| C2_GM001094 | 99.77 | 17.23 | 4.11 | 0.000934835 | 0.1505 | 246 | - | up |
| C2_GM002841 | 342.87 | 12.19 | 3.61 | 0.001019432 | 0.1552 | 543 | - | up |
| C2_GM001244 | 7853.35 | 10.32 | 3.37 | 0.001252291 | 0.1834 | 552 | hypothetical protein | up |
| C2_GM001146 | 3687.48 | 9.90 | 3.31 | 0.001518433 | 0.2060 | 186 | - | up |
| C2_GM002391 | 268.04 | 11.25 | 3.49 | 0.001569386 | 0.2060 | 1140 | CoA transferase | up |
| C2_GM000974 | 1039.43 | 9.09 | 3.18 | 0.002374531 | 0.2855 | 468 | hypothetical protein | up |
| C2_GM003148 | 381.86 | 9.47 | 3.24 | 0.002552231 | 0.2855 | 432 | hypothetical protein | up |
| C2_GM003334 | 328.60 | 9.44 | 3.24 | 0.002727836 | 0.2855 | 351 | hypothetical protein | up |
| C2_GM001145 | 5062.67 | 8.05 | 3.01 | 0.003446521 | 0.3456 | 591 | hypothetical protein | up |
| C2_GM001118 | 450.19 | 8.31 | 3.06 | 0.003947266 | 0.3720 | 756 | transferase 2, rSAM/selenodomain-associated | up |
| C2_GM003216 | 16881.64 | 7.72 | 2.95 | 0.003981007 | 0.3720 | 624 | malonic semialdehyde reductase | up |
| C2_GM001243 | 3910.03 | 7.59 | 2.92 | 0.004365198 | 0.3988 | 657 | hypothetical protein | up |
| C2_GM001144 | 7019.38 | 7.21 | 2.85 | 0.005246035 | 0.4622 | 633 | hypothetical protein ADIAG_00975 | up |
| C2_GM000075 | 391.72 | 7.77 | 2.96 | 0.005284594 | 0.4622 | 1365 | - | up |
| C2_GM002840 | 157.86 | 8.62 | 3.11 | 0.005575781 | 0.4730 | 525 | S26 family signal peptidase | up |
| C2_GM001027 | 16853.81 | 7.00 | 2.81 | 0.005850605 | 0.4730 | 1302 | Ammonium transporter 1 member 1 AtAMT1 | up |
| C2_GM000975 | 606.66 | 7.16 | 2.84 | 0.006422468 | 0.4889 | 279 | hypothetical protein ADIAG_00997 | up |
| C2_GM000977 | 521.06 | 7.16 | 2.84 | 0.006617877 | 0.4947 | 873 | (2Fe-2S)-binding protein | up |
| C2_GM004092 | 11262.18 | 6.71 | 2.75 | 0.006898139 | 0.4999 | 423 | MULTISPECIES: hypothetical protein | up |
| C2_GM002842 | 430.35 | 6.93 | 2.79 | 0.007788146 | 0.5427 | 525 | alternate signal-mediated exported protein, family | up |
| C2_GM003643 | 94103.84 | 6.24 | 2.64 | 0.008980555 | 0.5955 | 1095 | Stearoyl-CoA 9-desaturase | up |
| C2_GM000995 | 665.75 | 6.30 | 2.66 | 0.010101471 | 0.6489 | 318 | hypothetical protein | up |
| C2_GM001122 | 295.24 | 6.53 | 2.71 | 0.010692239 | 0.6703 | 930 | sugar ABC transporter permease | up |
| C2_GM000949 | 533.84 | 6.23 | 2.64 | 0.010924981 | 0.6703 | 1149 | glycosyl transferase | up |
| C2_GM000979 | 601.89 | 6.14 | 2.62 | 0.011285726 | 0.6823 | 525 | - | up |
| C2_GM003542 | 159.22 | 6.76 | 2.76 | 0.012357797 | 0.7187 | 870 | tryptophan 2,3-dioxygenase | up |
| C2_GM002015 | 1216.74 | 5.84 | 2.55 | 0.012412374 | 0.7187 | 645 | hypothetical protein | up |
| C2_GM000971 | 564.52 | 5.88 | 2.56 | 0.013271785 | 0.7373 | 234 | hypothetical protein | up |
| C2_GM000280 | 8698.10 | 5.56 | 2.48 | 0.013887908 | 0.7514 | 1374 | hypothetical protein | up |
| C2_GM001095 | 3452.93 | 5.44 | 2.45 | 0.015218518 | 0.8125 | 849 | HAD hydrolase | up |
| C2_GM001840 | 197.77 | 6.11 | 2.611 | 0.015487448 | 0.8163 | 480 | homoprotocatechuate degradation operon regulator, HpaR | up |
| C2_GM001117 | 353.97 | 5.75 | 2.52 | 0.015724752 | 0.8183 | 1374 | LmbE family protein | up |
| C2_GM000567 | 87.70 | 7.01 | 2.81 | 0.016555792 | 0.8253 | 543 | TetR family transcriptional regulator | up |
| C2_GM000998 | 5346.75 | 5.28 | 2.40 | 0.016862732 | 0.8253 | 615 | hypothetical protein | up |
| C2_GM002954 | 115.28 | 6.44 | 2.69 | 0.017469389 | 0.8276 | 984 | - | up |
| C2_GM000976 | 2722.06 | 5.24 | 2.39 | 0.017515244 | 0.8276 | 924 | beta 1,4 glucosyltransferase | up |
| C2_GM000978 | 11.51 | Inf | Inf | 0.018723097 | 0.8703 | 120 | - | up |
| C2_GM001128 | 2747.34 | 5.12 | 2.36 | 0.019100351 | 0.8725 | 417 | signal transduction protein with CBS domain | up |
| C2_GM004023 | 2223.84 | 5.05 | 2.34 | 0.020133799 | 0.8992 | 186 | CsbD family protein | up |
| C2_GM003238 | 184.61 | 5.64 | 2.50 | 0.020669073 | 0.8992 | 663 | TetR family transcriptional regulator | up |
| C2_GM003604 | 1525.34 | 5.01 | 2.32 | 0.021130462 | 0.8992 | 549 | hypothetical protein | up |
| C2_GM000999 | 3529.76 | 4.93 | 2.30 | 0.021584944 | 0.8992 | 459 | hypothetical protein | up |
| C2_GM001839 | 198.86 | 5.51 | 2.46 | 0.021603896 | 0.8992 | 1281 | MFS transporter | up |
| C2_GM001977 | 29062.29 | 4.90 | 2.29 | 0.021610165 | 0.8992 | 495 | ferritin | up |
| C2_GM001000 | 8551.81 | 4.80 | 2.26 | 0.023414091 | 0.9441 | 666 | hypothetical protein | up |
| C2_GM001446 | 435.54 | 5.05 | 2.34 | 0.023423847 | 0.9441 | 2076 | acyltransferase 3 | up |
| C2_GM002014 | 650077.76 | 4.73 | 2.24 | 0.024463675 | 0.9578 | 204 | MULTISPECIES: cold-shock protein | up |
| C2_GM002346 | 49.79 | 7.40 | 2.89 | 0.025023956 | 0.9705 | 1416 | - | up |
| C2_GM000288 | 9692.94 | 4.62 | 2.21 | 0.026718122 | 1.0000 | 228 | Antitoxin | up |
| C2_GM003768 | 165.15 | 5.24 | 2.39 | 0.027129516 | 1.0000 | 2643 | - | up |
| C2_GM001102 | 6726.26 | 4.59 | 2.20 | 0.027375383 | 1.0000 | 312 | hypothetical protein | up |
| C2_GM000973 | 3536.45 | 4.57 | 2.19 | 0.028112529 | 1.0000 | 639 | hypothetical protein | up |
| C2_GM001663 | 8370.74 | 4.53 | 2.18 | 0.028540104 | 1.0000 | 1272 | MFS transporter | up |
| C2_GM000214 | 186979.66 | 4.52 | 2.18 | 0.028557538 | 1.0000 | 1425 | type I glutamate--ammonialigase | up |
| C2_GM004087 | 1326.54 | 4.54 | 2.18 | 0.029683398 | 1.0000 | 756 | hypothetical protein | up |
| C2_GM002705 | 288099.65 | 4.46 | 2.16 | 0.029848431 | 1.0000 | 1524 | aldehyde dehydrogenase | up |
| C2_GM001661 | 1231.82 | 4.54 | 2.18 | 0.029931148 | 1.0000 | 1047 | glutamate-rich protein GrpB | up |
| C2_GM001952 | 5031.02 | 4.44 | 2.15 | 0.030742759 | 1.0000 | 564 | hypothetical protein | up |
| C2_GM002670 | 238.95 | 4.82 | 2.27 | 0.031049707 | 1.0000 | 1494 | tricarballylate dehydrogenase | up |
| C2_GM001659 | 159.67 | 5.04 | 2.33 | 0.031122771 | 1.0000 | 1629 | alkaline phosphatase | up |
| C2_GM001147 | 2585.10 | 4.37 | 2.13 | 0.032917948 | 1.0000 | 1032 | - | up |
| C2_GM000997 | 5222.80 | 4.33 | 2.12 | 0.033383355 | 1.0000 | 1173 | glutathione-dependent formaldehyde dehydrogenase | up |
| C2_GM000934 | 1057.44 | 4.33 | 2.12 | 0.035220095 | 1.0000 | 249 | hypothetical protein | up |
| C2_GM002240 | 4656.91 | 4.21 | 2.08 | 0.036691063 | 1.0000 | 1149 | pyruvate dehydrogenase (acetyl-transferring) E1 component subunit alpha | up |
| C2_GM003709 | 22269.92 | 4.19 | 2.07 | 0.037081507 | 1.0000 | 1296 | pyridine nucleotide-disulfide oxidoreductase | up |
| C2_GM001100 | 229.54 | 4.53 | 2.18 | 0.038108299 | 1.0000 | 192 | - | up |
| C2_GM001123 | 346.64 | 4.39 | 2.14 | 0.038302307 | 1.0000 | 849 | sugar ABC transporter permease | up |
| C2_GM002704 | 68753.48 | 4.13 | 2.05 | 0.038529551 | 1.0000 | 1026 | zinc-dependent alcohol dehydrogenase | up |
| C2_GM002839 | 89.70 | 5.15 | 2.36 | 0.039118922 | 1.0000 | 468 | - | up |
| C2_GM002637 | 371.843 | 4.33 | 2.11 | 0.03954921 | 1.0000 | 1545 | acyl-CoA synthetase | up |
| C2_GM000972 | 551.60 | 4.24 | 2.08 | 0.040069352 | 1.0000 | 363 | - | up |
| C2_GM000969 | 84.40 | 5.17 | 2.37 | 0.040153188 | 1.0000 | 270 | membrane protein | up |
| C2_GM002633 | 309.55 | 4.35 | 2.12 | 0.040463956 | 1.0000 | 864 | ABC transporter-like protein | up |
| C2_GM003766 | 867.81 | 4.16 | 2.06 | 0.04079386 | 1.0000 | 2379 | - | up |
| C2_GM000456 | 470.48 | 4.13 | 2.05 | 0.044196011 | 1.0000 | 960 | sulfate adenylyltransferase small subunit | up |
| C2_GM003161 | 2797.28 | 3.90 | 1.96 | 0.047663687 | 1.0000 | 633 | thymidylate kinase | up |
| C2_GM001116 | 390.10 | 4.06 | 2.02 | 0.047904907 | 1.0000 | 1068 | hypothetical protein | up |
| C2_GM001120 | 2036.90 | 3.89 | 1.96 | 0.04834103 | 1.0000 | 429 | hypothetical protein | up |
| C2_GM000866 | 3707.90 | 3.88 | 1.95 | 0.048384561 | 1.0000 | 912 | malonyl CoA-ACP transacylase | up |
| C2_GM001099 | 1612.91 | 3.88 | 1.96 | 0.049279531 | 1.0000 | 1473 | hypothetical protein | up |
| C2_GM002624 | 64.82 | 1.45 | 0.54 | 0.536990881 | 1.0000 | 1905 | vanillate O-demethylase oxidoreductase | increased |
| C2_GM002232 | 18835.83 | 1.48 | 0.57 | 0.549069121 | 1.0000 | 855 | catechol 1,2-dioxygenase | increased |
| C2_GM002345 | 445.27 | 2.17 | 1.12 | 0.260648376 | 1.0000 | 831 | catechol 2,3-dioxygenase | increased |
| C2_GM002649 | 297.31 | 1.76 | 0.82 | 0.415358640 | 1.0000 | 834 | protocatechuate 3,4-dioxygenase | increased |
| C2_GM003651 | 41330.93 | 3.03 | 1.59 | 0.101396933 | 1.0000 | 1284 | cytochrome P450 for pimelic acid formation for biotin biosynthesis | increased |
| C2_GM002227 | 711.78 | 0.02 | -5.45 | 3.9581E-06 | 0.00542 | 1308 | MFS transporter | down |
| C2_GM002314 | 1579.50 | 0.05 | -4.32 | 8.37492E-05 | 0.03825 | 858 | prenyltransferase | down |
| C2_GM002312 | 488.62 | 0.05 | -4.34 | 0.00011911 | 0.04897 | 402 | C50 carotenoid epsilon cyclase | down |
| C2_GM002315 | 3243.42 | 0.06 | -4.09 | 0.000156735 | 0.05858 | 1401 | FAD binding domain of DNA photolyase | down |
| C2_GM001716 | 2506.82 | 0.06 | -3.95 | 0.000241284 | 0.07085 | 2826 | FAD-linked oxidase | down |
| C2_GM002827 | 281.21 | 0.06 | -4.11 | 0.000312974 | 0.08245 | 990 | D-isomer specific 2-hydroxyacid dehydrogenase NAD-binding | down |
| C2_GM002826 | 708.82 | 0.07 | -3.90 | 0.000348259 | 0.08245 | 963 | hydroxymethylglutaryl-CoA lyase | down |
| C2_GM003946 | 28203.79 | 0.07 | -3.79 | 0.000361005 | 0.08245 | 1629 | cation acetate symporter | down |
| C2_GM002824 | 4363.39 | 0.08 | -3.64 | 0.000578677 | 0.11328 | 1446 | gluconate permease | down |
| C2_GM003945 | 36622.96 | 0.09 | -3.48 | 0.000885633 | 0.15050 | 345 | hypothetical protein | down |
| C2_GM002825 | 1344.90 | 0.09 | -3.50 | 0.000951812 | 0.15050 | 1245 | CoA-transferase | down |
| C2_GM002311 | 9524.81 | 0.10 | -3.35 | 0.001293697 | 0.18339 | 1623 | phytoene desaturase | down |
| C2_GM003447 | 743.52 | 0.10 | -3.35 | 0.001603214 | 0.20596 | 1323 | serine hydroxymethyltransferase | down |
| C2_GM002600 | 1128.56 | 0.10 | -3.28 | 0.00180699 | 0.22511 | 1884 | choline/carnitine/betaine transport | down |
| C2_GM002604 | 497.88 | 0.11 | -3.22 | 0.002526495 | 0.28546 | 1221 | formaldehyde dehydrogenase, glutathione-independent | down |
| C2_GM002309 | 12219.47 | 0.12 | -3.10 | 0.002645385 | 0.28546 | 912 | trans-hexaprenyltranstransferase | down |
| C2_GM002310 | 8069.21 | 0.12 | -3.08 | 0.002774811 | 0.28546 | 933 | squalene synthase HpnD | down |
| C2_GM002306 | 4119.99 | 0.12 | -3.09 | 0.002777562 | 0.28546 | 1473 | NAD-dependent dehydratase | down |
| C2_GM002226 | 4025.62 | 0.12 | -3.00 | 0.00353718 | 0.34622 | 840 | 1,6-dihydroxycyclohexa-2,4-diene-1-carboxylate dehydrogenase | down |
| C2_GM002233 | 2049.07 | 0.14 | -2.83 | 0.005851137 | 0.47298 | 261 | muconolactone delta-isomerase | down |
| C2_GM003446 | 358.60 | 0.13 | -2.93 | 0.005936292 | 0.47298 | 1218 | sarcosine oxidase subunit beta family protein | down |
| C2_GM002308 | 15793.38 | 0.14 | -2.80 | 0.00598273 | 0.47298 | 501 | MarR family transcriptional regulator | down |
| C2_GM001862 | 1541.32 | 0.14 | -2.81 | 0.006229505 | 0.48320 | 1266 | polyamine ABC transporter substrate-binding protein | down |
| C2_GM002307 | 4202.00 | 0.15 | -2.75 | 0.00693525 | 0.49991 | 813 | hypothetical protein | down |
| C2_GM001605 | 1896.65 | 0.15 | -2.76 | 0.007053035 | 0.49991 | 846 | high-affinity Fe2+/Pb2+ permease | down |
| C2_GM002313 | 36.39 | 0.06 | -3.96 | 0.008454246 | 0.57926 | 357 | C50 carotenoid epsilon cyclase | down |
| C2_GM002828 | 90.95 | 0.11 | -3.13 | 0.008744221 | 0.58930 | 987 | glycerate kinase | down |
| C2_GM002136 | 892.56 | 0.16 | -2.65 | 0.009942227 | 0.64877 | 1278 | hypothetical protein ADIAG_02145 | down |
| C2_GM001239 | 6424.77 | 0.17 | -2.57 | 0.010868167 | 0.67034 | 669 | sugar kinase | down |
| C2_GM000049 | 370.65 | 0.16 | -2.65 | 0.011653187 | 0.69429 | 1245 | aspartate aminotransferase family protein | down |
| C2_GM001931 | 218.85 | 0.16 | -2.68 | 0.013015538 | 0.73730 | 1026 | iron ABC transporter membrane protein | down |
| C2_GM003470 | 152.44 | 0.15 | -2.75 | 0.013196519 | 0.73730 | 1521 | putative cationic amino acid transporter APC family protein | down |
| C2_GM001240 | 2418.29 | 0.18 | -2.49 | 0.013890254 | 0.75135 | 1023 | sugar phosphate isomerase/epimerase | down |
| C2_GM003444 | 644.99 | 0.18 | -2.47 | 0.016129394 | 0.82527 | 2913 | glycine cleavage system T protein | down |
| C2_GM003386 | 289.47 | 0.17 | -2.53 | 0.016588127 | 0.82527 | 1527 | acetyl-CoA hydrolase/transferase family protein | down |
| C2_GM000919 | 18617.56 | 0.19 | -2.40 | 0.016758004 | 0.82527 | 2064 | NADH:flavin oxidoreductase/NADH oxidase | down |
| C2_GM000918 | 3215.86 | 0.19 | -2.39 | 0.01748129 | 0.82765 | 1305 | Rieske (2Fe-2S) domain-containing protein | down |
| C2_GM002234 | 2919.19 | 0.19 | -2.36 | 0.018841585 | 0.87031 | 1101 | mandelate racemase | down |
| C2_GM001863 | 113.03 | 0.16 | -2.61 | 0.021253824 | 0.89915 | 480 | transcriptional regulator | down |
| C2_GM000178 | 1548.28 | 0.20 | -2.32 | 0.021282111 | 0.89915 | 1494 | LytF | down |
| C2_GM001861 | 478.69 | 0.19 | -2.36 | 0.021653108 | 0.89915 | 1137 | polyamine ABC transporter substrate-binding lipoprotein | down |
| C2_GM001238 | 6716.04 | 0.21 | -2.27 | 0.02312313 | 0.94407 | 540 | - | down |
| C2_GM002603 | 5069.32 | 0.21 | -2.26 | 0.023905097 | 0.95412 | 1518 | flavohemoglobin | down |
| C2_GM000947 | 2020.88 | 0.21 | -2.26 | 0.024202005 | 0.95668 | 1122 | hypothetical protein | down |
| C2_GM002169 | 395.67 | 0.20 | -2.30 | 0.026148306 | 1.00000 | 672 | TetR family transcriptional regulator | down |
| C2_GM002305 | 940.16 | 0.22 | -2.21 | 0.028676174 | 1.00000 | 567 | transcriptional regulator | down |
| C2_GM001673 | 1062.59 | 0.22 | -2.20 | 0.029183662 | 1.00000 | 1677 | acetolactate synthase | down |
| C2_GM001759 | 59140.57 | 0.23 | -2.15 | 0.030674283 | 1.00000 | 1986 | acetyl-coenzyme A synthetase | down |
| C2_GM003078 | 3320.40 | 0.23 | -2.11 | 0.034039416 | 1.00000 | 1134 | - | down |
| C2_GM003647 | 1066.08 | 0.23 | -2.11 | 0.035356764 | 1.00000 | 1398 | MFS transporter | down |
| C2_GM001932 | 75.35 | 0.18 | -2.47 | 0.036966501 | 1.00000 | 990 | ferrichrome import ABC transporter permease | down |
| C2_GM001235 | 24467.08 | 0.24 | -2.07 | 0.037018677 | 1.00000 | 1062 | LacI family transcriptional regulator | down |
| C2_GM002265 | 48.99 | 0.16 | -2.69 | 0.037168393 | 1.00000 | 840 | 2,5-diketo-D-gluconic acid reductase | down |
| C2_GM003037 | 373.70 | 0.23 | -2.14 | 0.037509193 | 1.00000 | 1578 | periplasmic-binding protein AppA | down |
| C2_GM003947 | 237.48 | 0.22 | -2.19 | 0.037554839 | 1.00000 | 996 | - | down |
| C2_GM000327 | 3254.46 | 0.24 | -2.06 | 0.037912366 | 1.00000 | 1431 | glutamate synthase subunit beta | down |
| C2_GM002376 | 248.43 | 0.22 | -2.16 | 0.039564754 | 1.00000 | 843 | hypothetical protein | down |
| C2_GM002373 | 302.72 | 0.23 | -2.12 | 0.040479207 | 1.00000 | 1350 | MFS transporter | down |
| C2_GM002479 | 251.81 | 0.23 | -2.14 | 0.040988487 | 1.00000 | 1692 | hypothetical protein | down |
| C2_GM002381 | 693.94 | 0.24 | -2.05 | 0.042064335 | 1.00000 | 450 | - | down |
| C2_GM003621 | 3400.93 | 0.25 | -2.01 | 0.042376706 | 1.00000 | 939 | sodium-dependent transporter | down |
| C2_GM001731 | 1869.34 | 0.25 | -2.02 | 0.04239246 | 1.00000 | 1107 | phospho-2-dehydro-3-deoxyheptonate aldolase | down |
| C2_GM000046 | 2205.28 | 0.25 | -2.01 | 0.043437825 | 1.00000 | 978 | N-acetyl-gamma-glutamyl-phosphate reductase | down |

Table S11 Gene expression in the group of C2*△dyp* on glucose vs lignin

| **id** | **Base Mean** | **Fold Change(C/A)** | **log2 Fold Change** | **pval** | **padj** | **Length** | **Description** | **Level** |
| --- | --- | --- | --- | --- | --- | --- | --- | --- |
| C2_GM003651 | 27224.67 | 15.67 | 2.75 | 0.00228 | 1 | 1284 | cytochrome P450 for pimelic acid formation for biotin biosynthesis | up |
| C2_GM003649 | 5102.10 | 815.83 | 9.67 | 0.02204 | 1 | 1254 | nitrite reductase (NAD(P)H) large subunit | up |
| C2_GM001663 | 6015.77 | 721.32 | 9.49 | 0.02357 | 1 | 1272 | MFS transporter | up |
| C2_GM002569 | 4666.84 | 425.94 | 8.73 | 0.03254 | 1 | 690 | succinyl-CoA--3-ketoacid-CoA transferase subunit A | up |
| C2_GM002570 | 3745.65 | 341.66 | 8.42 | 0.03736 | 1 | 633 | succinyl-CoA--3-ketoacid-CoA transferase subunit B | up |
| C2_GM002435 | 1541.17 | 295.08 | 8.20 | 0.04202 | 1 | 1281 | cytochrome | up |
| C2_GM002624 | 47.67 | 2.39 | 1.26 | 0.70230 | 1 | 1095 | vanillate O-demethylase oxidoreductase | increased |
| C2_GM002232 | 10667.39 | 12.37 | 3.63 | 0.28146 | 1 | 855 | catechol 1,2-dioxygenase | increased |
| C2_GM002649 | 190.12 | 6.94 | 2.79 | 0.39819 | 1 | 834 | protocatechuate 3,4-dioxygenase | increased |
| C2_GM002345 | 398.73 | 2.02 | 1.02 | 0.74607 | 1 | 831 | catechol 2,3-dioxygenase | increased |
| C2_GM002686 | 172.42 | 1.13 | 0.18 | 0.95484 | 1 | 753 | Vanillate O-demethylase oxidoreductase | increased |
| C2_GM003184 | 284571.159 | 0.001 | -9.578 | 0.022037 | 1 | 1239 | monooxygenase | down |
| C2_GM003620 | 25103.796 | 0.002 | -8.681 | 0.032631 | 1 | 1209 | cystathionine beta-lyase | down |
| C2_GM003419 | 22147.037 | 0.003 | -8.594 | 0.033897 | 1 | 285 | hypothetical protein | down |
| C2_GM002199 | 130055.346 | 0.003 | -8.541 | 0.034562 | 1 | 1674 | putative ABC transporter substrate binding protein | down |
| C2_GM002141 | 250948.557 | 0.004 | -8.127 | 0.041333 | 1 | 546 | OsmC family protein | down |
| C2_GM003103 | 64985.023 | 0.004 | -8.063 | 0.042529 | 1 | 1638 | ABC transporter | down |
| C2_GM003104 | 51377.576 | 0.004 | -8.058 | 0.042643 | 1 | 1236 | acyl-CoA dehydrogenase | down |
| C2_GM003102 | 12894.854 | 0.004 | -8.006 | 0.043761 | 1 | 909 | oligopeptide ABC transporter permease | down |
| C2_GM003619 | 30208.405 | 0.004 | -7.987 | 0.044010 | 1 | 1338 | O-acetylhomoserine (thiol)-lyase | down |
| C2_GM003100 | 150527.237 | 0.004 | -7.797 | 0.047695 | 1 | 1659 | peptide ABC transporter substrate-binding protein | down |
| C2_GM003105 | 34531.717 | 0.005 | -7.713 | 0.049514 | 1 | 1419 | monooxygenase | down |
| C2_GM002142 | 21185.096 | 0.005 | -7.708 | 0.049680 | 1 | 1131 | hypothetical protein | down |

**Reference:**

Liu, T.T., Zhou, N.Y., 2012. Novel L-cysteine-dependent maleylpyruvate isomerase in thegentisate pathway of Paenibacillus sp. Strain NyZ101. J. Bacteriol. 194, 3987–3994.

Min, J., Chen, W.W., Hu, X.K., 2019. Biodegradation of 2,6-dibromo-4-nitrophenol byCupriavidus sp. Strain CNP-8: kinetics, pathway, genetic and biochemical characterization.J. Hazard. Mater. 361, 10–18.
